# CytoMAP: A Spatial Analysis Toolbox Reveals Features of Myeloid Cell Organization in Lymphoid Tissues

**DOI:** 10.1016/j.celrep.2020.107523

**Published:** 2020-04-21

**Authors:** Caleb R. Stoltzfus, Jakub Filipek, Benjamin H. Gern, Brandy E. Olin, Joseph M. Leal, Yajun Wu, Miranda R. Lyons-Cohen, Jessica Y. Huang, Clarissa L. Paz-Stoltzfus, Courtney R. Plumlee, Thomas Pö schinger, Kevin B. Urdahl, Mario Perro, Michael Y. Gerner

**Affiliations:** 1 Department of Immunology, University of Washington, Seattle, WA 98109, USA; 2 Seattle Children’s Research Institute, Seattle, WA 98109, USA; 3 Department of Pediatrics, University of Washington, Seattle, WA 98195, USA; 4 School of Medicine, University of Washington, Seattle, WA 98195, USA; 5 Roche Innovation Center Munich, Pharmaceutical Research & Early Development (pRED), Discovery Pharmacology, Nonnenwald 2, 82377 Penzberg, Germany; 6 Roche Innovation Center Zurich, Pharmaceutical Research & Early Development (pRED), Wagistrasse 10, 8952 Schlieren, Switzerland; 7 Lead Contact

## Abstract

Recently developed approaches for highly multiplexed imaging have revealed complex patterns of cellular positioning and cell-cell interactions with important roles in both cellular- and tissue-level physiology. However, tools to quantitatively study cellular patterning and tissue architecture are currently lacking. Here, we develop a spatial analysis toolbox, the histo-cytometric multidimensional analysis pipeline (CytoMAP), which incorporates data clustering, positional correlation, dimensionality reduction, and 2D/3D region reconstruction to identify localized cellular networks and reveal features of tissue organization. We apply CytoMAP to study the microanatomy of innate immune subsets in murine lymph nodes (LNs) and reveal mutually exclusive segregation of migratory dendritic cells (DCs), regionalized compartmentalization of SIRPa dermal DCs, and preferential association of resident DCs with select LN vasculature. The findings provide insights into the organization of myeloid cells in LNs and demonstrate that CytoMAP is a comprehensive analytics toolbox for revealing features of tissue organization in imaging datasets.

## In Brief

Stoltzfus et al. present CytoMAP, a spatial analytics platform that incorporates diverse statistical and visualization modules for analysis of cellular positioning, cell-cell interactions, global tissue structure, and heterogeneity of tissue microenvironments. Exploration of myeloid cell localization in lymph nodes reveals fundamental positional relationships between dendritic cell subsets and local vasculature.

## Graphical Abstract

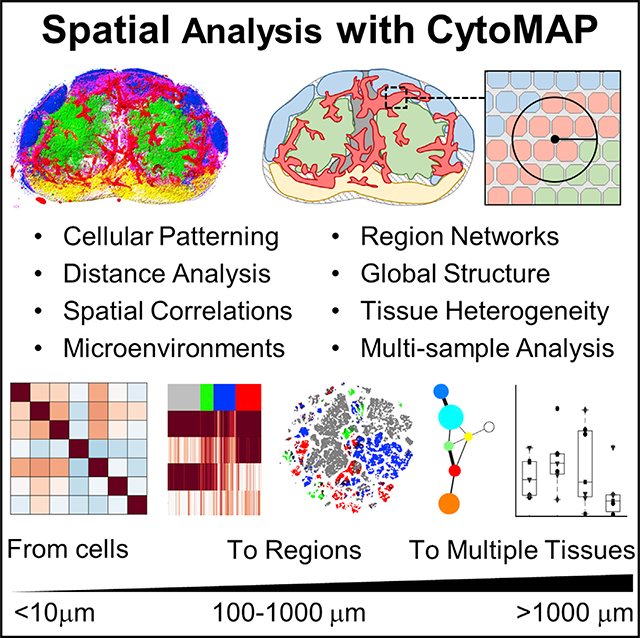

## INTRODUCTION

Recent advances in intravital microscopy and multiplexed imaging approaches have revealed that the spatial organization of cell populations in tissues is highly complex and intimately involved in diverse physiological processes, as well as in major pathological conditions, such as infections, autoimmunity, and cancer. For the immune system in particular, cellular positioning is critical for both cell homeostasis and generation of protective responses during infection or after vaccination (Eisenbarth, 2019; Groom, 2019; Qi et al., 2014). Within lymph nodes (LNs) alone, different subsets of dendritic cells (DCs) are spatially segregated within distinct tissue regions in a highly non-uniform fashion, which influences the sensitivity, kinetics, magnitude, and quality of the downstream adaptive immune response (Baptista et al., 2019; Gerner et al., 2012, 2015, 2017; Kissenpfennig et al., 2005; Kitano et al., 2016). Notably, advanced microscopy techniques have only recently revealed these findings in what were previously considered to be relatively well-studied organs, suggesting that further improvements in both microscopy and spatial analytics approaches can yield important insights into how complex biological systems operate.

This realization has inspired a number of emerging methods for highly multiplexed *in situ* cellular profiling (Eng et al., 2019; Gerner et al., 2012; Glaser et al., 2019; Gut et al., 2018; Li et al., 2019; Lin et al., 2015; Saka et al., 2019; Schürch et al., 2019; Vickovic et al., 2019; Winfree et al., 2017). These techniques generate panoptic datasets describing phenotypic, transcriptional, functional, and morphologic cellular properties while retaining information on the precise 2-dimensional (2D) or 3D positioning of cells within tissues. However, currently, there is a lack of accessible and simple-to-use tools for studying the complex multi-scale spatial relationships between different cell types and their microenvironments, for characterizing global features of tissue structure, and for understanding the heterogeneity of cellular patterning within and across samples. Existing approaches often utilize combinations of tools to reveal distance relationships between cells and tissue boundaries, utilize nearest neighbor and other statistical approaches to identify preferential associations among different cell types across relatively small tissue areas, or necessitate the extensive use of customized scripts (Caicedo et al., 2017; Coutu et al., 2018; Goltsev et al., 2018; Kraus et al., 2016; Mahadevan et al., 2017; Schapiro et al., 2017; Schürch et al., 2019). The lack of readily accessible and easy-to-use analytics tools has hampered the ability of biologists with access to high-dimensional imaging technologies to obtain an in-depth understanding of the spatial relationships of cells and their surrounding tissue microenvironments within quantitative imaging datasets.

Here,wedevelopeda user-friendly,spatialanalysismethod,the histo-cytometric multidimensional analysis pipeline (CytoMAP), which utilizes diverse statistical approaches to extract and quantify information about cellular spatial positioning, preferential cell-cell associations, and global tissue structure. We implemented CytoMAP as a comprehensive toolbox in MATLAB specifically designed to analyze datasets generated with existing quantitative approaches that already incorporate information on cell phenotype, morphology, and location. CytoMAP markedly simplifies spatial analysis by grouping cells into local neighborhoods, which can then be rapidly analyzed to reveal complex patterns of cellularcomposition,region structure, and tissueheterogeneity. The CytoMAP platform incorporates multiple modules for analysis, including: machine-learning-based data clustering, cellular position correlation, distance analysis, visualization of tissue patterning through dimensionality reduction, region network mapping, and 2D or 3D region reconstruction. Analysis with CytoMAP reveals and quantitates 2D or 3D tissue architecture, local cell composition, and cell-cell spatial networks, as well as the interconnectedness of tissue regions. CytoMAP also facilitates sample-to-sample comparison, allowing exploration of structural and compositional heterogeneity across samples and diverse experimental conditions. Furthermore, CytoMAP can be utilized for the analysis of positionally resolved data generated with diverse methods and across scales of various lengths, allowing integration into various disciplines.

We validate the capabilities of CytoMAP by investigating innate and adaptive cell organization in steady-state murine LNs, as well as in disease-associated tissues, including solid tumors and Mycobacterium tuberculosis (Mtb)-infected lung granulomas (Cadena et al., 2017; Gern et al., 2019; Keren et al., 2018; Plumlee et al., 2020). Our analyses recapitulate previous descriptions of the cellular microenvironments within these tissues and identify previously unappreciated features of myeloid cell organization in LNs. Specifically, we reveal predominant localization of migratory SIRPa dermal DCs (dDCs) within the lower cortical ridge of LNs, as well as preferential association of LN-resident DCs with select LN blood vessels (Girard et al., 2012; Moussion and Girard, 2011; Ochiai et al., 2014; Tussiwand et al., 2015).

## RESULTS

### CytoMAP Workflow

**Figure 1.**
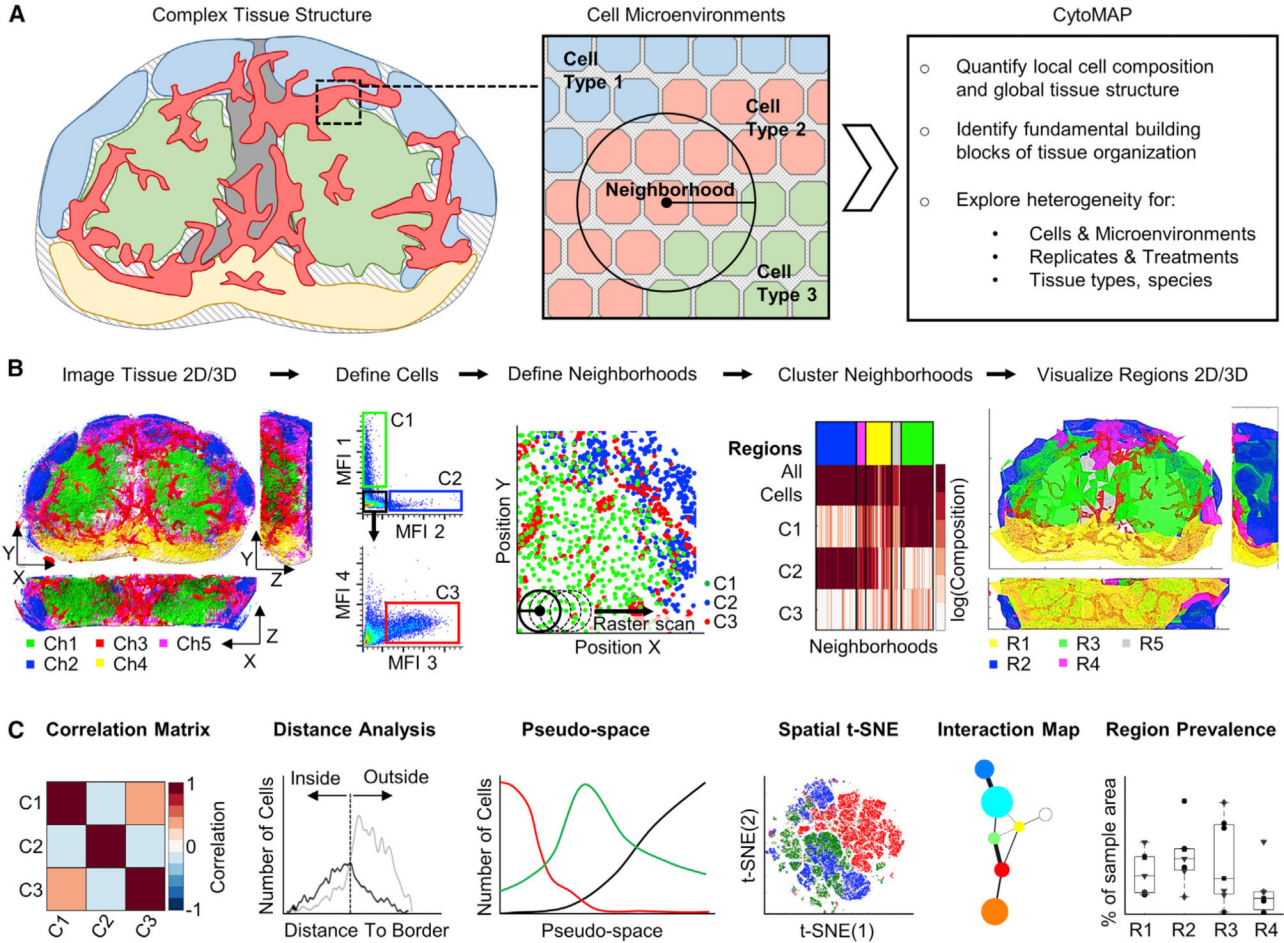
Workflow and Features of CytoMAP (A) CytoMAP is designed to extract quantitative information on cellular localization and composition within tissue regions, revealing how local cell microenvironments form global tissue structure, as well as allowing comparison of intra- and inter-sample tissue heterogeneity. (B) The workflow starts with multi-parameter imaging of either thin sections or large 3D tissue volumes. Next, hierarchical gating of cell objects is used to annotatedistinct cell subsets, which are passed into CytoMAP for analysis. CytoMAP segments these spatial datasets into individual neighborhoods and uses clustering algorithms to define similar groups of neighborhoods, or tissue “regions,” which are explored and spatially reconstructed in 2D or 3D space. (C) CytoMAP contains multiple tools to quantify and visualize the tissue architecture, including analysis of spatial correlations between different cell types,investigation of distance relationships of cells with architectural landmarks, analysis of neighborhood heterogeneity within individual tissues or across multiple samples, and quantitative visualization of tissue architecture.

Tissues are composed of different cell types that group together into local neighborhoods. Similar neighborhoods extend further across large distances to generate tissue regions, and distinct spatial combinations and associations of regions collectively form the overarching tissue structure. Based on this concept, CytoMAP utilizes information on cell type and position to phenotype local neighborhoods and reveal how their spatial distribution leads to the generation of global tissue architecture (Figure 1A).

To accomplish this, the acquired phenotypic properties and positional information of individual cell objects are first passed to CytoMAP, in which they are spatially subdivided into local neighborhoods of user-defined dimensions (Figure 1B). The generated neighborhoods contain information on the cell composition and density and the expression of specific molecules, as well as data on any additional structural or functional parameters. These parameters are next passed to a self-organizing map (SOM) that clusters the neighborhoods into groups (Gut et al., 2018; Jiang et al., 2010; Kohonen, 1990; Vesanto et al., 2000). The clustered neighborhoods represent areas within the tissue with similar cellular composition and are, thus, defined here as tissue “regions,” which are denoted by the different colors in the top of the example heatmap in Figure 1B. This heatmap allows direct visualization of the cellular composition of the neighborhoods (columns on the heatmap) in the identified regions and shows the relative prevalence of the different regions within the imaged sample, as denoted by the size of the region color bars. The neighborhoods are next spatially remapped and color coded by region. This allows direct visualization of the size and structure of the different regions within the tissue. Finally, the multiple visualization and quantification techniques incorporated into CytoMAP are used to form a more complete understanding of the spatial properties of cells, neighborhoods, and regions within tissues (Figure 1C). These include cell-cell spatial correlation analysis, distance mapping, and dimensionality reduction tools, as well as region interconnectedness and prevalence analyses, among others, which collectively provide a comprehensive platform for spatial analysis.

### CytoMAP Quantifies Well-Defined Tissue Structure in LNs

**Figure 2.**
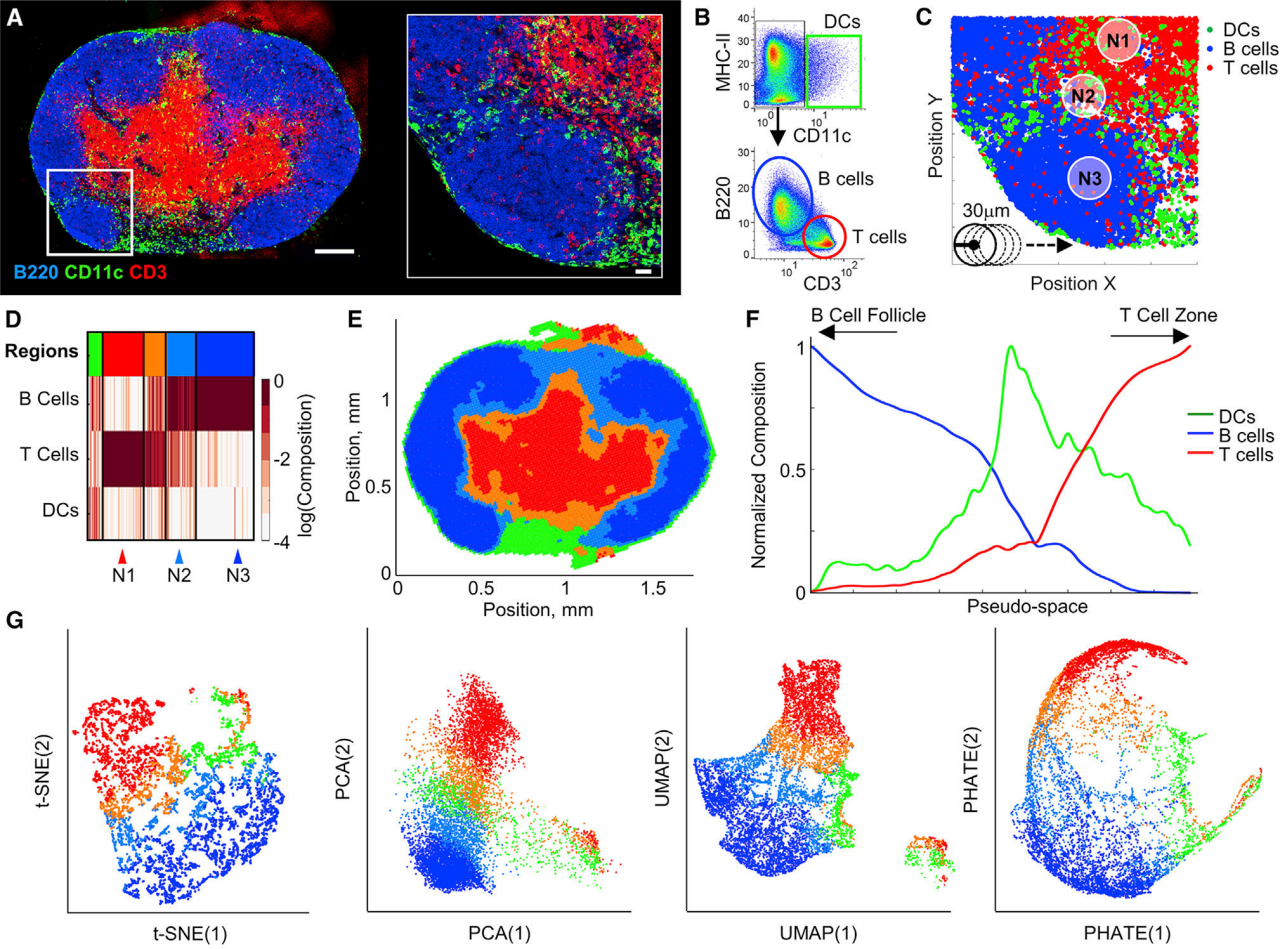
CytoMAP Identifies Major Features of LN Tissue Structure (A) Confocal image and zoom-in image of a LN section from a C57BL/6 mouse, adoptively transferred with 10^6 naive OT-II CD4+ T cells and immunized with OVA plus alum. Overview image scale bar, 200 μm; zoom-in scale bar, 30 μm. Only select channels are shown. (B) Histo-cytometry plots of cell object MFI for different channels demonstrating the gating used for identification of the indicated immune cell populations. (C) Positional plot of cell data from (B) (area matches the zoom-in image in A). CytoMAP was used to calculate the number of cells in 30-μm-radius neighborhoods (denoted by the circles in the bottom left), which were raster scanned as denoted by the arrow. (D) Heatmap of the neighborhood composition (percentage of each cell phenotype per neighborhood) after SOM clustering. Individual clusters, or “regions,” aredenoted by the color bar at the top of the graph. Arrowheads at the bottom highlight specific neighborhoods. (E) Region color-coded positional plot of the neighborhoods from (D). (F) Pseudo-space plot with the neighborhoods sorted based on B cell composition (sorted to the left) and T cell composition (sorted to the right). (G) Dimensionality reduction plots of the neighborhoods in which the standardized numbers of cells and total MFI of all channels were used for the dimensionalityreduction. t-SNE, PCA, UMAP, and PHATE were all calculated for the same input neighborhoods, which are color coded based on region type from (D). For this experiment, an imaging volume of 0.03 mm^3^, 139,399 cells, and 11,328 neighborhoods were analyzed.

We first validated the CytoMAP workflow by analyzing murine LN tissues, which have well-defined cellular organization (Qi et al., 2014). To accomplish this, a 20-mm-thick section of a draining LN from a C57BL/6 mouse was stained with a panel of directly conjugated antibodies against distinct innate and adaptive immune populations (Table S1) and imaged using a confocal microscope. The image in Figure 2A indicates the staining of the tissue with markers for B cells (B220), DCs (CD11c), and T cells (CD3). Images were next analyzed by histo-cytometry (Gerner et al., 2012; Li et al., 2017, 2019), with the individual cells first segmented in 3D and then with the cell objects’ mean channel fluorescent intensity (MFI) and position (x,y, and z) information imported into FlowJo for hierarchical gating of three primary cell types: T cells, B cells, and CD11c-expressing cells (primarily DCs) (Figure 2B). Next, the positional data on these cell populations were imported into CytoMAP for further processing. In CytoMAP, the cells were subdivided into 30-mm-radius neighborhoods using the “raster scan neighborhoods” function, which digitally raster scanned a cylindrical window with the user-defined radius over the dataset (Figure 2C). This neighborhood radius was chosen empirically, as it provided an optimal balance of spatial granularity and processing speed to reveal major features of cellular organization for this sample (Figure S1A), and it was also consistent with dispersion distances of secreted cytokines (Oyler-Yaniv et al., 2017). A SOM was next used to cluster these neighborhoods based on their cellular composition but not their tissue location. The number of regions was determined using the Davies-Bouldin criterion, which calculates the ratio of within-cluster to between-cluster distances (Figure S1B) (Davies and Bouldin, 1979). The heatmap in Figure 2D indicates the cell composition (rows) of the individual neighborhoods (columns) and the cluster/region (top color bar) to which they were assigned. This analysis identified tissue regions that were primarily composed of B cells, T cells, DCs, or those with mixed cellular composition. These regions were next visualized by plotting the positions of the color-coded neighborhoods (Figure 2E): this demonstrated reconstruction of the original image, revealing the localization of computationally defined B cell follicles (blue), the deep T cell zone (red), the outer T zone paracortex and the T-B border (orange), and the LN medullary and subcapsular regions (green).

In addition to discreet region definitions, we implemented pseudo-space to simplify visualizing the continuous region transitions within tissues (Figure 2F). Pseudo-space allows the user to sort neighborhoods by the absolute number, or composition, of different cell types within the neighborhoods and plots the neighborhoods in this sorted order on a linear axis. This provides a qualitative picture of how different cell types change in their composition across the neighborhoods along this user-defined dimension. Pseudo-space visualization showed that, as the percentage of B cells in the neighborhoods declined, the percentage of T cells increased (Figure 2F), mirroring what was observed in the original image (Figure 2A). In the transitional area between the B cell- and T cell-rich neighborhoods, there was an increase in DCs, likely corresponding to the increased abundance of DCs in the medullary and paracortical regions of the LN.

To further explore neighborhood heterogeneity, we used severalestablishedalgorithms:t-DistributedStochasticNeighbor Embedding(t-SNE),principal-componentanalysis(PCA),uniform manifold approximation and projection (UMAP), and potential of heat-diffusion for affinity-based transition embedding (PHATE), which all reduce the complexity of neighborhoods into a twodimensional plot (van der Maaten and Hinton, 2008; McInnes et al., 2018; Meehan et al., 2020; Moon et al., 2019). Instead of individual cells, the cellular composition and biomarker expression of the neighborhoods, but not their position, were used for the dimensionality reduction. These analyses revealed a complex structure in the LN dataset with clearly demarcated but also interconnected clusters of neighborhoods (Figures 2G and S1D). Color coding of neighborhoods based on regions, as defined in Figure 2D, revealed clear association of the distinct region types with the different clusters, suggesting that both SOM clustering and dimensionality reduction methods are capable of identifying cellular organization within tissues. This was confirmed using manual gatingwithin thet-SNE plot (Figure S1E),and spatialmapping (Figure S1F) of the t-SNE clusters, which accurately reconstructed the global tissue architecture as well as identified image artifacts (black color-coded gate in Figures S1E and S1F).

Dimensionality reduction also demonstrated the inter-connected nature of the regions, showing neighborhoods from the B cell follicle and T cell zone regions being connected with one another by neighborhoods assigned to the paracortical region (orange in Figures 2G and S1D). This is likely due, in part, to smoothing effects from raster scanning the neighborhoods in steps of half the defined radius, so that neighborhoods partially overlap. However, this structure also captured features of the actual tissue organization, identifying the paracortical T-B border regions where T cells and B cells are in sufficient spatial proximity to be included in the same neighborhood (Figure 2C, neighborhood N2). Furthermore, we noted an increased cluster structure when performing t-SNE analysis of non-normalized data (Figure S1D). To understand how such a structure was generated, we visualized the mean channelintensitiesperneighborhoodwithheatmaps(FigureS1D). This demonstrated substantial variation in the channel intensities across the different t-SNE sub-clusters, suggesting that additional neighborhood separation is driven by the degree of local channel signal, likely representing local cellular abundance.

We next used network analysis to interrogate the interconnectedness of the regions by calculating the percentage of the region borders that are shared with other regions (Figure S1G). Mapping of these border relationships revealed that the T cell zone region (red) is connected to the B cell region (blue) via the paracortical region (orange), recapitulating the dimensionality reduction analysis and the original image (Figures 2A, 2G, and S1G). Changing the size of the neighborhoods in this simplistic example did not change the interconnectedness of the regions (Figure S1G).

### CytoMAP Analysis of Tumor Microenvironments

**Figure 3.**
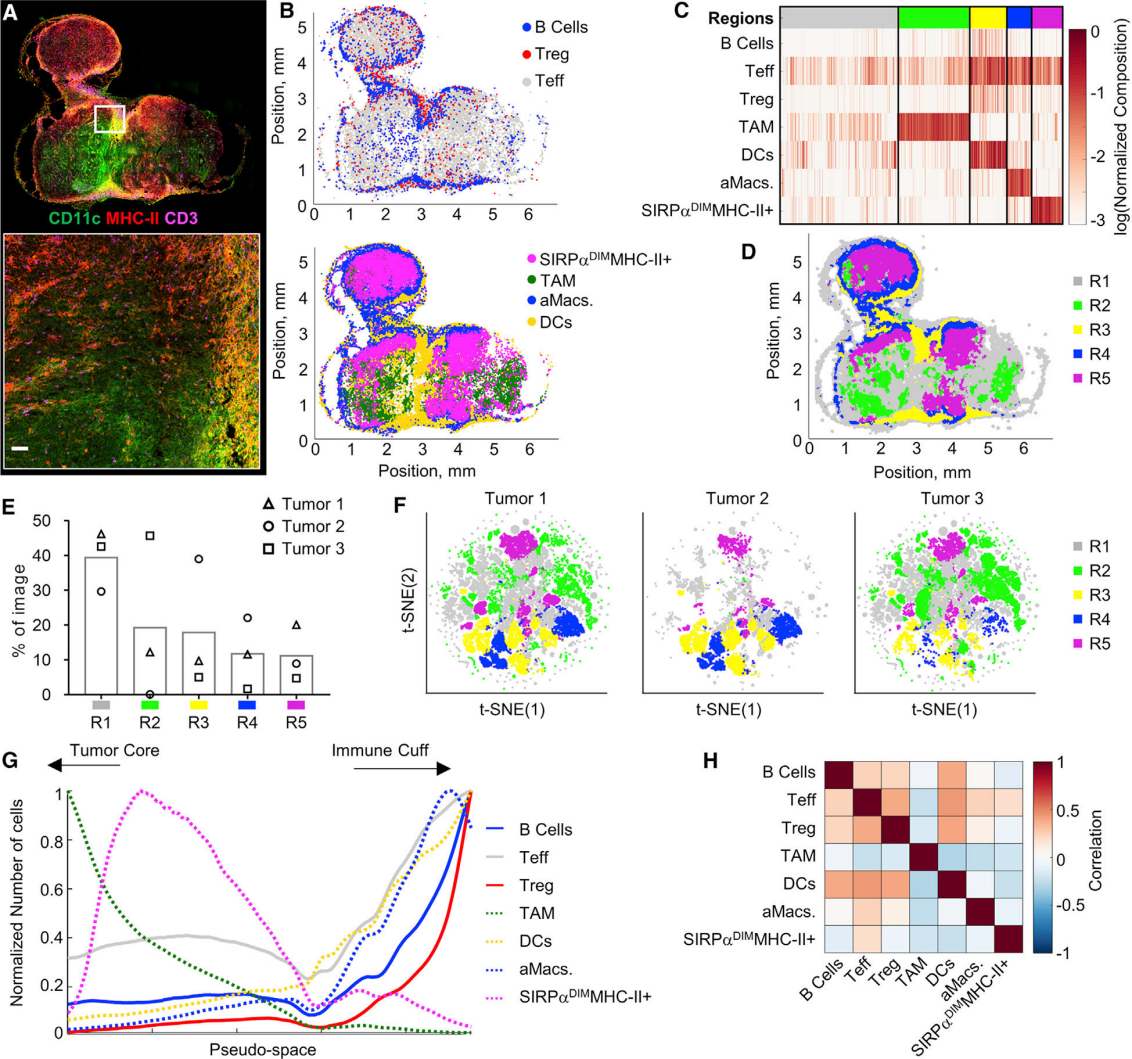
CytoMAP Analysis of a Murine Colorectal Tumor Sample (A) Confocal image of a representative 20-μm-thick CT26 tumor section isolated 9 days after subcutaneous inoculation. Zoom-in image shows a region in the tumor periphery. Main image scale bar, 500 μm; zoom-in scale bar, 50 μm. (B) Positional plot of the lymphocyte (top) and myeloid cell (bottom) populations. (C) Heatmap of the normalized immune cell composition of regions defined by SOM clustering of 50-μm-radius neighborhoods from all imaged samples. (D) Positional plot of neighborhoods color coded by region defined in (C). (E) Region prevalence plot showing the percentage of the neighborhoods from each sample in each region. (F) t-SNE plots of the neighborhoods from all three samples, color coded based on region type. (G) Pseudo-space plot with TAM-rich neighborhoods sorted to the left and DC-rich neighborhoods sorted to the right. (H) Heatmap of the Pearson correlation coefficients of the number of cells per neighborhood across the imaged tumor samples. For this experiment, 3 tumor samples with a total imaging volume of 1.8 mm^3^ and a total of 102,354 myeloid cells, 68,801 lymphocytes, and 192,785 neighborhoods were analyzed.

We next tested CytoMAP by exploring the distribution of immune cells in more complex tissue types. For this, we imaged cross-sections of murine CT26 colorectal tumors stained with a panel of markers to detect various innate and adaptive immune populations (Figure 3A). Histo-cytometry was used to gate on CD3^+^Foxp3^−^T cells (putative T effector cells [Teffs]), CD3^+^

Foxp3^+^ T regulatory cells (Tregs), B220^+^CD3 B cells, MHC-IICD64^+^ myeloid cells (putative monocyte-derived tumor-associated macrophages [TAMs]), CD11cSIRPa^+^MHC-II^+^ myeloid cells (putative activated macrophages [aMacs]), CD11c^+^MHC-II^+^ DCs, and MHC-II^+^ SIRPa^DIM^ myeloid cells (Figure S2A) (DeNardo and Ruffell, 2019; Jahchan et al., 2019; Veillette and Chen, 2018). Although these limited markers provide inexact cell annotations, the spatial distribution of the identified populations revealed substantial intra-tumoral heterogeneity and striking compartmentalization of the tumor tissue into areas enriched with different immune cell subsets (Figure 3B). SOM clustering of neighborhoods identified tissue regions preferentially associated with specific myeloid cell populations (Figures 3C and S2B). These region definitions were also largely consistent across permutations of the number of samples used to train the clustering algorithm (Figures S2B and S2C). Spatial visualization of the neighborhoods revealed discrete tumor zones composed of relatively segregated region types (Figure 3D). For example, region R3 was predominantly composed of DCs and Teff cells and was primarily localized to the outer periphery of the tumor (Figures 3C, 3D, and S2B), resembling the lymphocytic cuff observed in colorectal and other cancers (Keren et al., 2018). If neighborhoods from either tumor 1 or tumor 2, which have increased portions of this peripheral region (Figures 3E and 3F), were excluded from the training dataset, the Davies-Bouldin criterion identified 7 instead of 5 regions, effectively splitting the DC-rich R3 region into three separate sub-regions (Figures S2B and S2C). In contrast, region R2 was dominantly composed of TAMs and was consistently identified and localized within the deeper portions of the tumor (Figures 3C, 3D, and S2B).

Further pseudo-space analysis showed an increased presence of T cells, B cells, and DCs in the tumor periphery and their relative exclusion from the TAM-rich tumor core (Figure 3G). This also demonstrated that pseudo-space analysis can effectively reduce cellular patterning to a single dimension and aid in visualization of highly complex tissues. These relationships were next quantified using the Pearson correlation coefficients of the number of cells per neighborhood (Figures 3H and S2D). Such cell-cell spatial correlation analyses help reveal which cell populations preferentially associate with one another or, conversely, avoid one another. This revealed a positive spatial correlation of T and B cells with DCs and aMacs and a negative correlation with TAMs. Notably, the negative correlation of Teff cells and TAMs indicates that, while Teff cells are generally capable of infiltrating the tumor tissue, they are not enriched in the areas dominantly populated with TAMs and, thus, lack the ability to infiltrate the deep tumor nests in this cancer model. Together, this diverse collection of visualization and quantification metrics generates a detailed picture of the tissue structure and cellular relationships within tumors.

### CytoMAP Reveals Organization of Immune Cells in Mtb Granulomas

**Figure 4.**
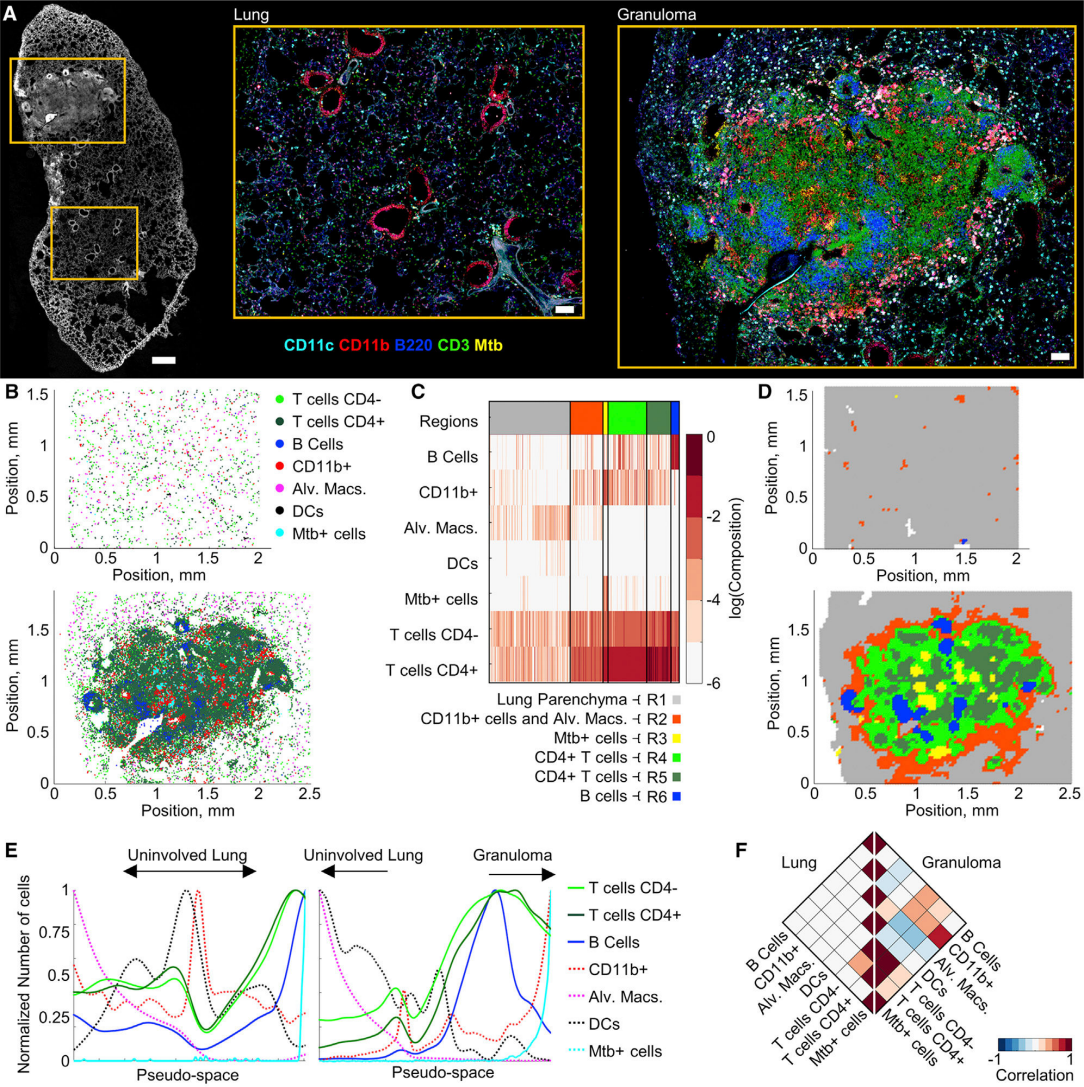
CytoMAP Analysis of Mtb-Infected Lung Granuloma (A) Confocal image of a 20-μm-thick section from a Mtb-infected murine lung sample. The left image shows multiple imaged channels overlaid in white. Scale bar, 500 μm. Zoom-in images show separately acquired regions of interest of an uninvolved lung area and the Mtb granuloma. Zoom-in scale bars, 100 μm. (B) Positional plots of the immune cell subsets in the uninvolved (top) versus granuloma (bottom) lung areas shown in (A). (C) Heatmap of SOM-clustered, 50-μm-radius neighborhoods. (D) Positional plots of the neighborhoods defined in (C), color coded by region, within the uninvolved (top) versus granuloma (bottom) lung areas. (E) Pseudo-space plots of the uninvolved (left) versus granuloma (right) lung neighborhoods after sorting for Alv. Macs (sorted to the left) and Mtb^+^ cells (sorted to the right). (F) Rotated half-heatmaps of the Pearson correlation coefficients of the number of cells within the neighborhoods across the uninvolved lung (left) and thegranuloma (right). For the uninvolved lung region, an imaging volume of 0.05 mm^3^, 36,194 cells, and 4,725 neighborhoods were analyzed. For the granuloma lung region, an imaging volume of 0.07 mm^3^, 140,453 cells, and 7,350 neighborhoods were analyzed.

In addition to tumors, previous studies have demonstrated structured organization of immune cells within Mtb-infected pulmonary granulomas (Cooper, 2009; Gern et al., 2019; Kahnert et al., 2007; Plumlee et al., 2020; Ramakrishnan, 2012). Therefore, we tested the ability of CytoMAP to explore this organization in a 20-mm lung section from a mouse infected with aerosolized Mtb. We observed the formation of a discrete Mtb lung granuloma with complex cellular patterning (Figure 4A). Two regions of interest, one of the unaffected lung and one of the Mtb granuloma (Figure 4A), were imaged at high resolution, and histo-cytometry was used to gate on CD4^+^ and CD4 T cells, B cells, CD11b^+^ myeloid cells, alveolar macrophages (Alv. Macs), DCs, and Mtb^+^-infected cells (Figures 4B and S3A) (Cohen et al., 2018; Gibbings et al., 2017). SOM neighborhood clustering in CytoMAP revealed distinct region types composed of different immune cell types (Figures 4C, S3B, and S3C). Remapping of the region color-coded neighborhoods demonstrated that the neighborhoods enriched in Mtb-infected and CD11b^+^ myeloid cells (region R3) were primarily located in the deep center of the granuloma (Figure 4D). These infected regions were surrounded by neighborhoods containing high concentrations of T cells (regions R4 and R5), which were further surrounded by regions associated with uninfected myeloid cells and Alv. Macs (region R2). There were also segregated B cell-rich neighborhoods (region R6) (Figure 4D), which are reminiscent of B follicles and tertiary lymphoid structures. Pseudo-space analysis hoods also appeared excluded from the central granuloma showed that, within the granuloma, T cell- and B cell-rich neigh- core, recapitulating the primary image (Figure 4A). These relaborhoods were concentrated just outside of the core infected tionships were further quantified using Pearson correlation coefMtb^+^ cells (Figure 4E). The Alv. Macs and DC-rich neighbor- ficients of the numbers of cells in the neighborhoods (Figures 4F and S3D). In this analysis, the left half of the heatmap, representing uninvolved lung, showed no strong correlations between the different cell types. In contrast, the right half of the heatmap, representing the granuloma, showed a positive spatial correlation between the Mtb^+^-infected cells and the CD11b^+^ myeloid cells, a weaker correlation with the surrounding T cells, and a negative correlation with Alv. Macs. These findings are consistent with previous observations of immune cell organization in Mtb granulomas describing the partial segregation of CD4^+^ T cells from Mtb-infected cells and the formation of tertiary lymphoid structures (Kahnert et al., 2007). Collectively, these data indicate that CytoMAP is capable of quantitative spatial analysis of highly complex tissue structures across diverse organs and disease settings.

### CytoMAP Maps Myeloid Cell Organization in SteadyState LNs

**Figure 5.**
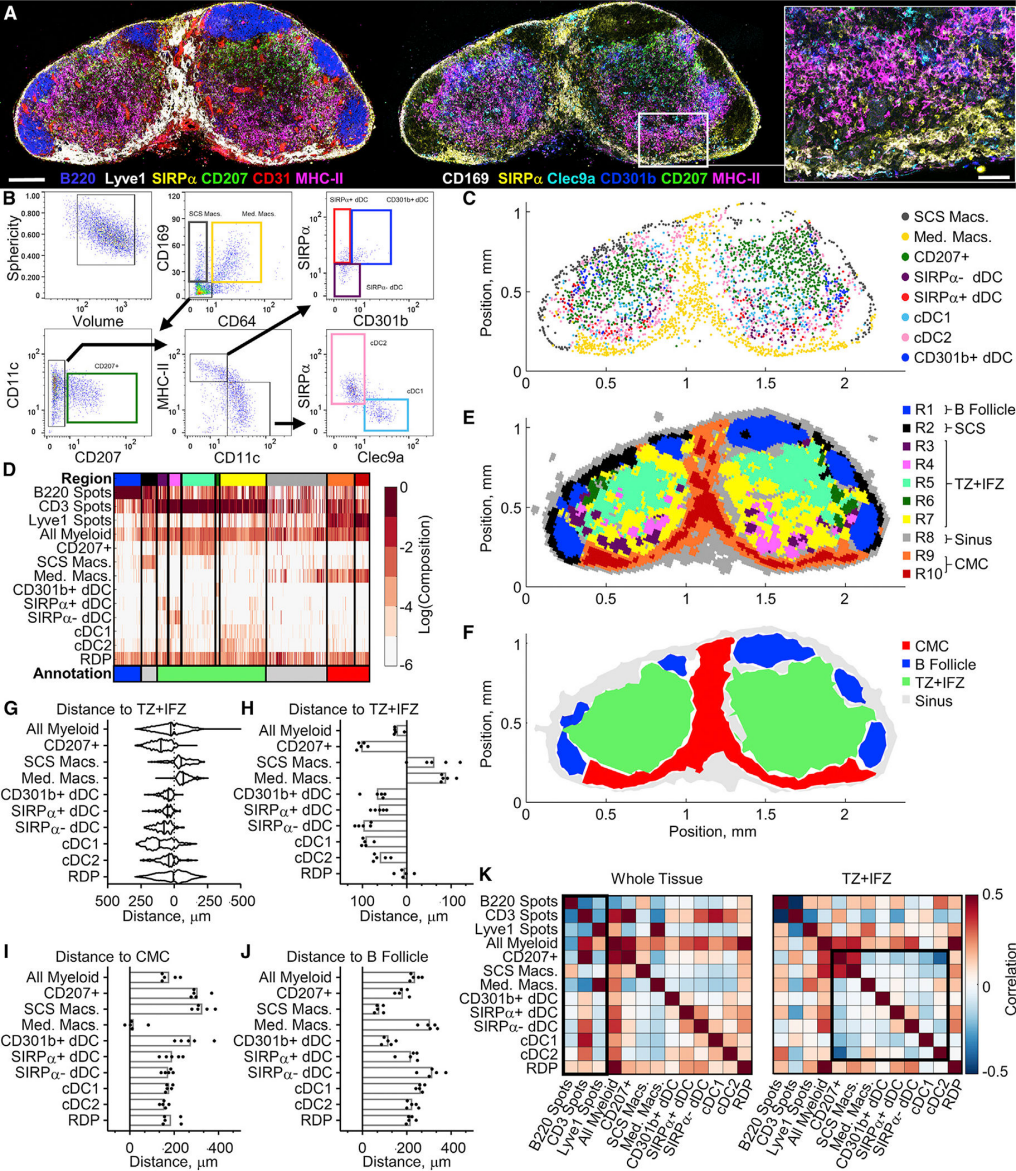
CytoMAP Reveals Diverse Patterns of Myeloid Cell Organization in LNs (A) Confocal image of a representative steady-state, non-draining LN section from an immunized C57BL/6 mouse. Scale bar, 200 μm; right zoom-in scale bar, 50 μm. (B) Histo-cytometry gating scheme used to annotate cell subsets. (C) Positional plots of the myeloid cell subsets (D) Heatmap of the cellular composition of 30-μm-radius neighborhoods showing regions as defined by SOM clustering (top color bar) and regions defined by manual annotation (bottom color bar). (E) Positional plot of the LN neighborhoods as color coded by the region type (top color bar in D). (F) Positional plot of surfaces generated on the manually annotated regions (bottom color bar in D). (G) Violin plot showing the number of cells as a function of distance to the border of the TZ+IFZ annotated region for the representative LN sample. Distances tothe left of zero represent cells inside the region; distances to the right of zero represent cells outside the region. (H) Plot of the distances of cells to the TZ+IFZ region, in which each dot represents the mean distance of the indicated cell population in an individual LN sample (n = 5). (I) Mean distances of indicated cells to the CMC region. (J) Mean distances of indicated cells to the B follicle region. (K) Plot of the Pearson correlation coefficients between the number of indicated cells per neighborhood using either all tissue neighborhoods (left) or only the TZ+IFZ region neighborhoods (right). Correlations were averaged over the sample cohort (n = 5) from one experiment. For this experiment, 5 LNs with an average imaging volume of 0.023 mm^3^ and an average of 6,611 myeloid cells, 84,347 spots, and 12,708 neighborhoods were analyzed per sample. Data are representative of at least two independent experiments.

Finally, we used CytoMAP to investigate the distribution of myeloid cell subsets in steady-state LNs, which have been previously shown to have intricate patterns of cellular localization (Eisenbarth, 2019; Gerner et al., 2012, 2015; Qi et al., 2014). To this end, 20-mm sections from a cohort of LN samples were stained with a 12-plex antibody panel to detect distinct DC and macrophage subsets, as well as to visualize T cells, B cells, and blood and lymphatic stromal cells as landmark reference structures. Tissues were imaged (Figure 5A), and distinct cell populations were identified by histo-cytometry (Figures 5B and S4A) (Crozat et al., 2011; Merad et al., 2013). Basic spatial remapping of these populations in CytoMAP qualitatively validated previous findings on the location of different DC and macrophage subsets (Figure 5C). In particular, we observed that subcapsular sinus and medullary macrophages (SCS Macs and Med Macs, respectively) localized to the outer LN periphery and medullary regions, respectively. Resident cDC1s and cDC2s also exhibited previously established spatial patterns, with the resident cDC2s preferentially localizing in the LN periphery and the resident cDC1s more evenly distributed within the T cell zone (Figures 5C and S4B) (Brewitz et al., 2017; Gerner et al., 2012; Kitano et al., 2016). Similarly, visualization of the migratory DC subsets confirmed previous findings, with CD207^+^ cells (Langerhans cells and migratory cDC1s) (Merad et al., 2013) located within the central T cell zone and the CD301b^+^ and SIRPa^+^ dDC subsets located in regions bordering the B cell follicles and in the lower cortical ridge, respectively (Figures 5C and S4B) (Gerner et al., 2012; Kissenpfennig et al., 2005; Kitano et al., 2016; Kumamoto et al., 2013; Sokol et al., 2018). In addition, histo-cytometry analysis revealed a recently described population of migratory SIRPa dDCs (Ochiai et al., 2014; Tussiwand et al., 2015). This migratory DC subset appeared predominantly localized in the lower cortical ridge bordering the LN medulla (Figures 5A, 5C, and S4B).

To aid in tissue region classification, additional spot objects were generated on the landmark channels (i.e., CD3, B220, and Lyve1 for the T cell zone, B cell follicles, and lymphatics, respectively), and the positional data from these landmark objects were passed into CytoMAP. In addition, with the “generate random points” function in CytoMAP, a population of randomly distributed points (RDPs) was defined (Figure S4B). Heatmap visualization of clustered neighborhoods showed 10 distinct region types (see the color bar at the top of Figure 5D) that were enriched in different cell types. Positional remapping of the region color-coded neighborhoods recapitulated the general features of the primary image (Figures 5E and S5A–S5D) and demonstrated segregation of the DC and macrophage subsets into distinct spatial compartments, corroborating previous observations (Gerner et al., 2012). Region visualization further confirmed the predominant localization of the SIRPa migratory dDCs (region R4) in the lower cortical ridge (Figures 5E, S4B, and S5A). Additional visualization of neighborhood clustering using UMAP analysis supported these region annotations while also revealing how these regions were spatially associated with one another across samples (Figure S5D).

We next used CytoMAP to manually annotate the regions based on landmark spot abundance (Figures 5D, S4B, and S5C). This resulted in new composite regions, color annotated at the bottom of the heatmap in Figure 5D, with region R1 corresponding to B cell follicles (blue), region R2 corresponding to the SCS (gray), regions R3–R7 corresponding to the T cell zone and interfollicular zones (TZ+IFZ; green), region R8 corresponding to the sinus (gray), and regions R9 and R10 corresponding to the cortico-medullary cords (CMCs; red) (Figures 5D, 5F, and S5C). Next, using the “make surface” function in CytoMAP, we built surfaces around the neighborhoods in these annotated groups and then calculated the distances of the myeloid cells to the borders of these surfaces (Figures 5G and S5E). The distance to each region border for all cells in a single sample (Figures 5G and S5E), or averaged over all cells for multiple samples (Figures 5H–5J and S5F), showed that most DCs were distributed within the TZ+IFZ, while the macrophages were positioned in either the SCS or the CMCs. This analysis also confirmed preferential localization of resident cDC2s in closer proximity to the CMCs and B cell follicles, compared to the more heterogeneous distribution of resident cDC1s within the LN (Figures 5I and 5J). CD301b^+^ DCs were found in close proximity to the B cell follicles, as previously described (Kumamoto et al., 2013), while the SIRPa migratory dDCs were located distally from the B cell follicles and in close proximity to the CMCs (Figures 5I, 5J, and S5F).

The discrete clustering and segregation of different myeloid cell types (Figures 5D and 5E) also indicated that the DC subsets are distributed in spatially non-overlapping patterns. To explore this further, we calculated the Pearson correlation coefficients of the number of cells per neighborhood, averaged over all of the samples, either for the whole samples or only within the TZ+IFZ regions (Figures 5K and S5G). Whole-tissue correlation analysis showed that all DC populations were positively correlated with CD3 spots and negatively correlated with Lyve1 spots and Med Macs, indicating that, on average, most DCs are located in the T cell zone (Figure 5K, left). Analysis of the TZ+IFZ compartment revealed that migratory DC populations (i.e., CD207^+^ DCs, CD301b^+^ dDCs, SIRPa^+/^ dDCs) were, in general, negatively correlated with one another or displayed little spatial correlation (Figure 5K, right), thus providing quantitative support for their spatial segregation in LNs. Together, these analyses highlight the ability of CytoMAP to delineate complex patterns of cellular organization into quantitative metrics.

### CytoMAP Quantifies Myeloid Cell Associations with LN Blood Vessels

**Figure 6.**
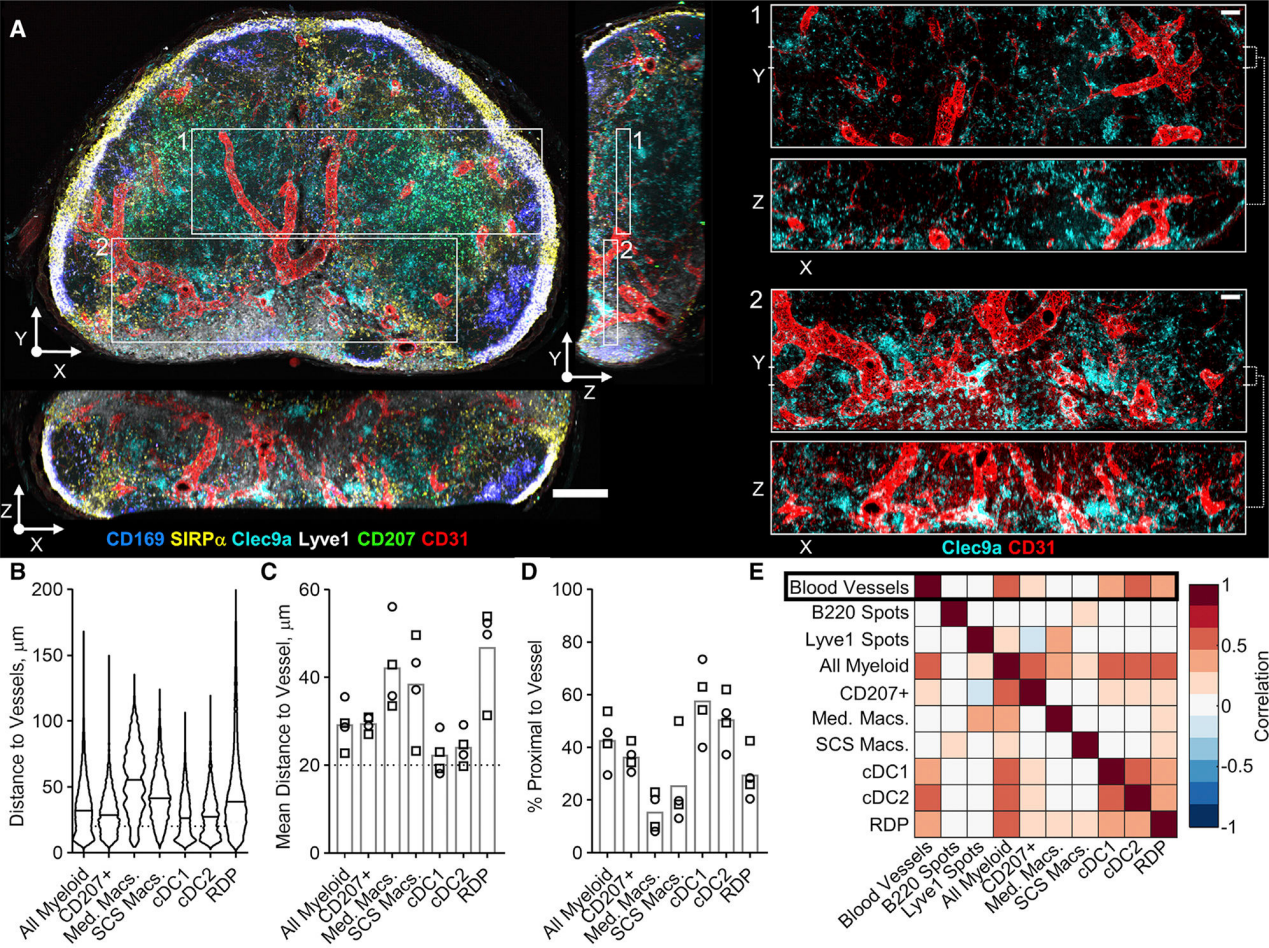
CytoMAP Analysis of 3D LNs Reveals Preferential Proximity of DCs with Vasculature (A) Representative confocal image of a 500-μm-thick, Ce3D-cleared, steady-state LN slice. Main image scale bar, 200 μm; zoom-in scale bars, 50 μm. (B) Violin plot comparing the distances of the indicated cells to the nearest blood vessels within a representative LN sample. (C) Mean distances of cell populations to the nearest blood vessel, with each symbol representing an individual LN sample. Circles and squares representsamples from two independent experiments. Cells below the dotted line at 20 μm are considered proximal to vasculature. (D) Percentage of proximal cells for the LN samples shown in (C). (E) Heatmap indicating the Pearson correlation coefficients, averaged across all imaged samples, between the number of different cell or landmark object typesper 50-μm-radius neighborhood. For this experiment, an average imaging volume of 0.93 mm^3^ and an average of 39,139 myeloid cells, 36,049 blood vessel objects, 98,279 spots, and 36,049 neighborhoods were analyzed per sample. Data represent four samples from two independent experiments.

A qualitative examination of imaged LN sections also revealed that some DC populations were highly proximal to LN blood vessels. Given the established role of DCs in the homeostatic maintenance of LN vasculature (Girard et al., 2012; Moussion and Girard, 2011), we examined the relative distribution of DC subsets with respect to blood vessels. To minimize the sampling error associated with thin section imaging, we performed volumetric imaging of stained and Ce3D optically cleared 500-mm-thick slices of steady-state murine LNs (Figure 6A) (Li et al., 2017, 2019). Intriguingly, qualitative examination of the imaged LNs revealed close association of Clec9a^+^ DCs with CD31^+^ vascular endothelial cells, and many of these DCs encapsulated large segments of blood vessels (Figure 6A; Video S1). In contrast, CD207^+^ cells (migratory cDC1 and Langerhans cells) (Merad et al., 2013) appeared less associated with the LN vasculature. Clec9a can be expressed on both LN-resident and migratory cDC1 subsets (Crozat et al., 2011). In these specific volumetric datasets, we did not achieve optimal major histocompatibility complex class II (MHC-II) staining for robust discrimination of migratory versus resident DCs. However, in thin-section imaging, Clec9a labeling is primarily associated with CD11c^HIGH^MHC-II^DIM^SIRPa cells, corresponding to resident cDC1s (Figures S4C–S4F). Additionally, Clec9a staining does not greatly overlap with CD207 signal, which is associated with migratory DCs (Figures 5A and 6A). Thus, while some of the detected Clec9a^+^ DCs in the volumetric datasets may be attributed to additional myeloid populations (Figure 6A), they largely represent LN-resident cDC1s. To quantitate these observations, we used histo-cytometry to identify various myeloid subsets and passed their data into CytoMAP (Figures S6A and S6B) (Li et al., 2017, 2019). To provide positional information on CD31^+^ blood vessels, we generated segmented surface objects on the CD31 channel and imported these objects’ data into CytoMAP. As mentioned earlier, positional information of B220^+^ B cell and Lyve1^+^ lymphatic sinus landmark spots were also included. In addition, RDPs were defined throughout the 3D LNs for comparison. The distances for the different myeloid cell subsets, or RDPs, to the nearest blood vessel object were next calculated (Figure 6B). Visualization of the individual cell distances to the closest blood vessel demonstrated that, while there was substantial heterogeneity within a given cell population, both resident cDC1s and cDC2s (both resident and migratory) were, on average, located in close proximity to blood vessels (Figures 6B–6D). This was in contrast to more distal relationships for most migratory CD207^+^ DCs, Macs, or RDPs. Pearson correlation coefficients, for the number of cells per 50-mm-radius neighborhood, further showed that, in contrast to migratory DCs, resident cDC1s and cDC2s were both positively correlated with blood vessels (Figures 6E and S6C).

**Figure 7.**
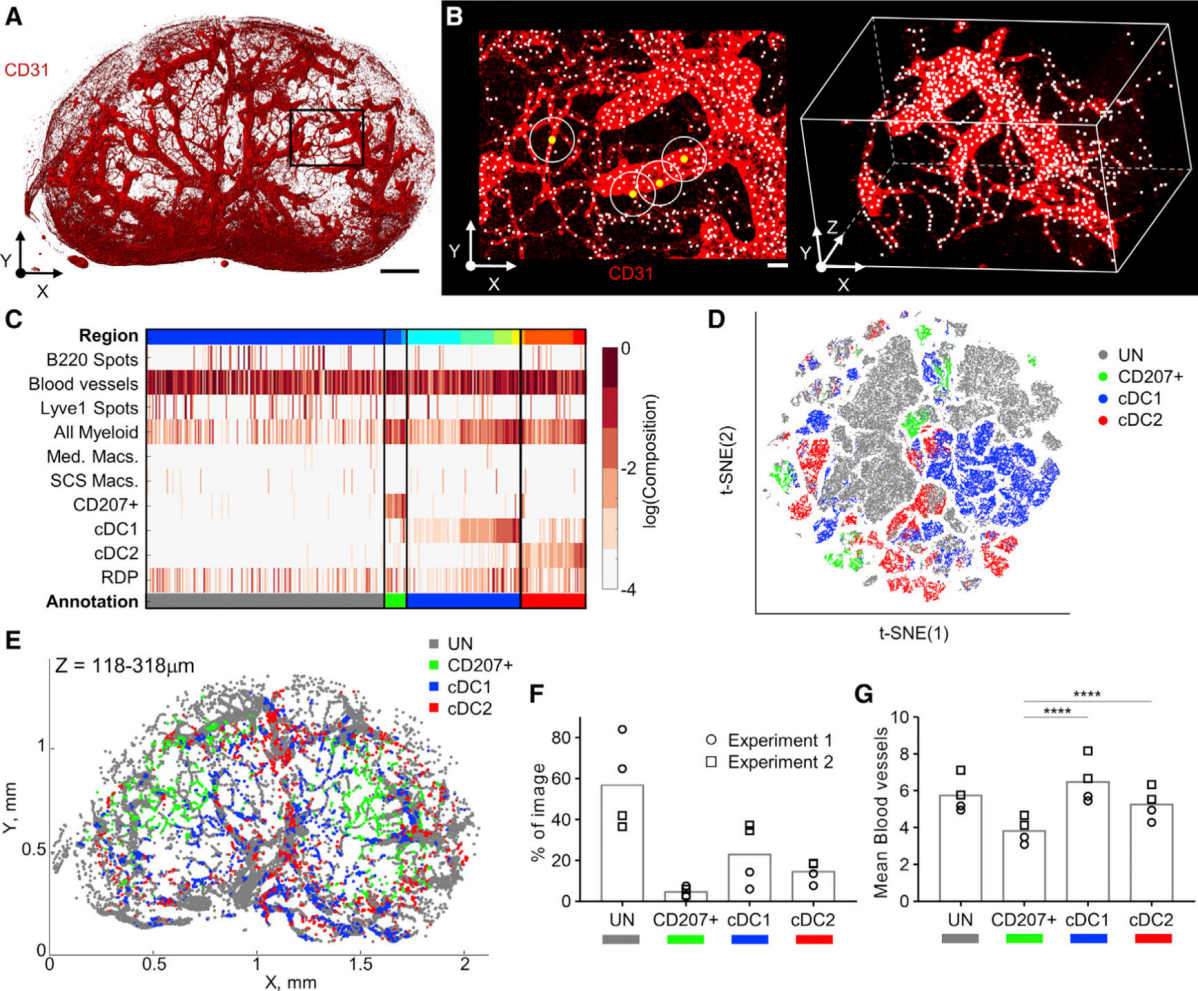
Individual Blood Vessel Branches in LNs Demonstrate Selective Associations with Specific DC Subsets (A) Confocal image demonstrating the CD31-labeled vascular networks in the same representative LN sample as presented in Figure 6A. Scale bar, 200 μm. (B) Zoom-in of the region in (A) (denoted with a rectangle), indicating the centers of CD31^+^ blood vessel objects (white dots). The circles with yellow dots represent spherical-object-centered neighborhoods with a radius of 20 ¼m. Scale bar, 20 μm. (C) Heatmap of the myeloid cell composition for the vessel-centered neighborhoods after SOM clustering. The top color bar indicates color-coded regions ofneighborhoods. The bottom color bar indicates manually annotated regions. (D) t-SNE plot of neighborhoods from all samples color coded by region from (C). (E) Positional plot of the vascular neighborhoods in a 200-μm-thick virtual Z section, color coded based on the annotations in (C). (F) Region prevalence plot showing the percentage of the neighborhoods from each sample in each region. (G) Mean number of blood vessel objects in each neighborhood for each region showing the size of the vessels associated with each cell type. ****p < 0.0001, ascalculated by repeated-measures one-way ANOVA with multiple comparisons. Data represent the same four samples from two independent experiments used in Figure 6.

To explore the cellular microenvironments associated with the complex vascular networks in LNs (Figure 7A), we next utilized the “cell centered neighborhoods” function in CytoMAP, which, instead of raster scanning neighborhoods, generates neighborhoods centered on the positions of the selected objects. This analysis approach allows a more focused interrogation of the cellular relationships for the selected population of interest. Here, 20-mm-radius spherical neighborhoods were centered on the CD31^+^ vascular objects to selectively identify vascular-associated myeloid cells (Figure 7B). This is demonstrated in Figure 7B, in which white dots demarcate the centers of all blood vessel objects, with the example 2D projections of the spherical neighborhoods (circles) shown to surround individual objects (yellow dots). All vascular neighborhoods were next clustered using the SOM algorithm, with the number of regions determined using the Davies-Bouldin criterion (Figure S7A). This clustering separated the vascular neighborhoods into several distinct types (Figures 7C, top color bar, 7D, and S7B), which were next manually grouped into four major blood vessel phenotypes based on the local DC composition (Figure 7C, bottom color bar; Figure S7B). This clustering revealed that, while many neighborhoods were not closely associated with any given myeloid cell type, unassociated (UN), large portions of the vascular neighborhoods were preferentially associated with either the resident cDC1s or the cDC2s (Figures 7C–7F). A smaller number of neighborhoods were also associated with CD207^+^ migratory cells. t-SNE analysis confirmed these SOM-based annotations and the differential clustering of neighborhoods based on distinct myeloid cell associations (Figure 7D). Importantly, 3D spatial remapping of the different vascular subtypes in CytoMAP revealed that large segments of vascular branches were almost exclusively associated with either the resident cDC1s, cDC2s, or CD207^+^ DCs, with little local spatial intermixing (Figures 7E and S7C–S7E; Video S2). In addition, analysis of the mean number of blood vessel objects per neighborhood indicated that the cDC1 and cDC2 cells associate with significantly larger blood vessels as compared to the CD207^+^ DCs, likely representing high endothelial venules versus capillary associations, respectively (Figure 7G). Together, these data indicate that discrete vascular branch segments are differentially associated with distinct myeloid cell partners.

## DISCUSSION

The importance of quantitative imaging and spatial analysis has emerged across a diverse spectrum of biological disciplines at different length scales, from the localization of single molecules within individual cells to the organization of cells across whole organs. Various technologies now allow spatially resolved highcontent detection of diverse probe types, from protein and oligonucleotide imaging to lipid visualization (Colomb et al., 2019; Gerner et al., 2012; Glaser et al., 2019; Goltsev et al., 2018; Gut et al., 2018; Keren et al., 2018; Li et al., 2017, 2019; Schapiro et al., 2017; Schulz et al., 2018; Stoltzfus et al., 2015). These approaches provide an unprecedented level of detail in biological processes, and as the imaging area and number of analytes increase, the development of tools for analyzing these increasingly complex and voluminous datasets is critical. Here, we develop a comprehensive analysis platform, CytoMAP, capable of robust spatial analysis of cellular organization within tissues. CytoMAP harnesses the power of unsupervised clustering, dimensionality reduction, and advanced data visualization to expand the utility of spatially resolved cellular profiling. CytoMAP integrates data on cellular phenotypes and position to identify unique neighborhoods and regions within tissues and organs, which provides the ability to interrogate complex spatial patterns across heterogeneous samples in a matter of minutes (Table S2). Here, we utilized CytoMAP to explore and quantify the organization of immune cells and microenvironments within lymphoid tissues as well as in tumor and Mtb-infected lung samples. Our results show both well-established and previously unappreciated cellular organization within these tissues. In particular, our findings identify the localization of SIRPa migratory dDCs within the lower cortical ridge of the T cell zonebordering the LN medulla as well as reveal preferential associations of LN-resident DC populations with blood vessel networks in distinct LN compartments.

CytoMAP reduces data complexity in two major ways. First, CytoMAP treats complex cell objects as individual points, each possessing information on the positional, morphological, and phenotypic cellular characteristics averaged over their respective 3D segmented cellular bodies. Second, CytoMAP defines raster scanned, or cell-centered, local neighborhoods across the tissue, effectively binning data on many similarly positioned cells into single data points. The ability to manipulate the size of the neighborhoods facilitates exploration of overarching tissue structure or detailed investigation of the hyperlocal cellular microenvironments and cell-cell proximities. However, over-binning the cells into very large neighborhoods may lead to excessive data smoothing and loss of neighborhood heterogeneity. Conversely, decreasing the size of the neighborhood size below the size of reasonable cellular networks is likely to generate noisier data output as well as require extensive computational resources. Thus, care and empirical testing should be used when choosing this parameter.

After raster scanning, similar neighborhoods are grouped together via clustering, which computationally defines unique tissue regions within highly complex datasets with minimal user input. Once clustered, CytoMAP provides tools for exploring the cellular composition and relative prevalence of the tissue regions within and across samples, as well as for visualizing these regions in 2D or 3D space. Interaction network maps provide additional detail into how the regions are spatially interconnected with one another to generate global tissue structure. Several dimensionality reduction tools in CytoMAP allow the user to reveal cellular patterning across the tissues as well as examine the heterogeneity of individual or multiple samples. Finally, neighborhood-based correlation analysis facilitates the exploration of how different cell populations are spatially correlated with one another, revealing preferential cellular associations or mutual exclusivity of different cell types. Together, the combination of comprehensive and flexible analytical approaches built into a user-friendly interface makes CytoMAP a powerful toolbox for the exploration of complex cellular spatial relationships within large multiplexed spatially resolved datasets.

One area where spatial analysis tools may provide substantial benefit is in cancer research, in which isolated tumor biopsies have poorly understood cellular organization but still possess substantial prognostic value. To test CytoMAP with such heterogeneous tissues, we explored the organization of immune cells in CT26 tumor samples. This analysis identified several hallmark features of tumor architecture, including a lymphocytic cuff as well as centralized positioning of TAMs, which corroborates previous histological studies (Keren et al., 2018). CytoMAP also revealed moderate-to-high baseline infiltration of CT26 tumors by effector T cells, suggesting that this cancer model should be susceptible to checkpoint blockade therapy, which is in line with published data (Kim et al., 2014; Swart et al., 2016). Interestingly, in addition to different myeloid cell types localizing to distinct regions within the tumor, t-SNE plots of the neighborhoods suggest potential additional layers of cellular organization that should be explored in future studies. Visualizing these relationships across whole tumor cross-sections revealed substantial intra-tumoral heterogeneity with respect to the local composition of myeloid cells and lymphocytes. As expected, heterogeneity in cellular composition is also observed between different samples, even with the use of a syngeneic cancer model and genetically identical mice. Thus, sufficient sampling must be performed to provide adequate representation of the different region subtypes within individual samples as well as across cohort studies. This suggests that accurate risk/prognostic assessment of neoplastic tissues may benefit from access to larger tissue samples in addition to punch core biopsies.

As an additional test of CytoMAP, we analyzed cellular organization within granuloma structures in a murine Mtb-infected lung sample. We observed the partial segregation of infiltrating CD4^+^ T cells from Mtb-infected myeloid cells and the formation of B cell aggregates. The presence of these distinct tissue regions as identified by CytoMAP’s clustering algorithm largely agrees with previous studies describing the organization of Mtb granulomas (Cadena et al., 2017), suggesting that CytoMAP presents a promising avenue for investigating the spatial organization of cells in complex inflamed and infected tissues. Given the extensive heterogeneity of granulomas across and even within single individuals (Flynn et al., 2011), analysis of additional samples is essential for verification of which regions and tissue structures are conserved across distinct granulomas. Follow-up work to dissect this issue is currently underway (Gern et al., 2019; Plumlee et al., 2020).

As a final test of CytoMAP, we analyzed the organization of myeloid cells in steady-state murine LNs. Consistent with previous observations, we found extensive spatial segregation and a high degree of mutual exclusivity for many of the DC subsets within LNs. In addition, we have identified the spatial distribution of a recently described SIRPa migratory dDC population (Ochiai et al., 2014; Tussiwand et al., 2015). These migratory dDCs appear predominantly localized within the lower cortical ridge bordering the LN medulla and, together with the locally positioned SIRPa^+^ dDCs, generate a prominent cuff-like cellular aggregate. In addition, analysis of resident cDC1s and cDC2s supports previous findings, with preferential localization of cDC2s in the LN periphery and more heterogeneous distribution of resident cDC1s across the T cell zone (Baptista et al., 2019; Brewitz et al., 2017; Gerner et al., 2012, 2015, 2017; Kitano et al., 2016; Leal et al., 2019). Importantly, we found that both resident cDC1 and cDC2 subsets are highly associated with LN blood vessels and are preferentially associated with distinct vascular trees, with little local intermixing. Although previous studies have shown that DCs can associate with blood vessels during inflammation (Bajé noff et al., 2003; Dasoveanu et al., 2016), our findings reveal that this normally occurs in the steady state and may thereby promote homeostatic maintenance of LN vasculature (Girard et al., 2012; Moussion and Girard, 2011). Our findings also suggest that blood vessels could provide positional cues to guide resident cDC1 and cDC2 distribution in LNs. While previous studies have identified a role for the G-protein coupled receptor 183 in guiding resident cDC2 positioning and survival in lymphoid tissues (Gatto et al., 2013; Yi and Cyster, 2013), little is known about the regulation of resident cDC1 localization in LNs. Our study provides hints to a potential mechanism regulating their distribution and will require further follow-up. Finally, our findings suggest that DC subset associations with distinct vascular branches could also influence blood endothelial cell biology, potentially promoting the recently described heterogeneity of LN blood endothelial cells (Takeda et al., 2019; Veerman et al., 2019).

In sum, here we develop a user-friendly, comprehensive, and broadly applicable spatial analysis toolbox for analysis of 2D or 3D quantitative imaging datasets, which excels at utilizing high-dimensional imaging data to reveal complex tissue features based on cellular phenotypic heterogeneity and spatial patterning. Our technology allows cross-sample interrogation of spatial cellular relationships and tissue architecture, and it reveals intra- as well as inter-sample heterogeneity. In this early implementation, CytoMAP has already provided useful insights into the organization of myeloid cells in lymphoid tissues, revealing the localization of SIRPa migratory dDCs as well as identifying preferential associations of resident DCs with select LN vasculature. Phenotyping neighborhoods with unsupervised clustering reveal distinct regions that are both biologically relevant and consistent across multiple samples. Together, this indicates that CytoMAP is a valuable tool for the rapid identification of key cellular networks and tissue structure and for revealing the fundamental building blocks of tissue organization.

## STAR+METHODS

### LEAD CONTACT AND MATERIALS AVAILABILITY

Further information and requests for resources and reagents should be directed to and will be fulfilled by the lead contact, Michael Gerner (gernermy@uw.edu). This study did not generate new unique reagents.

### EXPERIMENTAL MODEL AND SUBJECT DETAILS

#### Mice

For the experiments described in Figure 2, 5–7, 6–10 week old male and female B6.SJL and C57BL/6J mice were obtained from The Jackson Laboratory and kept in specific pathogen–free conditions at an Association for Assessment and Accreditation of Laboratory Animal Care–accredited animal facility at the University of Washington, South Lake Union campus. All procedures were approved by the University of Washington Institutional Animal Care and Use Committee. The influence of the sex of the mice was not assessed.

For the data presented in Figure 3, 6–10 week old female BALB/c mice were obtained from Charles River (Sulzfeld, Germany) and were housed in specific pathogen-free conditions. The animal facility was accredited by the Association for Assessment and Accreditation of Laboratory Animal Care and all animal studies were performed in accordance with the guidelines outlined by the Federation for Laboratory Animal Science Association and the German Animal Welfare law. The animal study was approved by and done under license obtained from the Government of Upper Bavaria (Regierung von Oberbayern; license number: ROB-55.22532.Vet_03–15-41).

For the experiment described in Figure 4, an 8 week old female C57BL/6J mouse was obtained from The Jackson Laboratory and housed in specific pathogen-free conditions at Seattle Children’s Research Institute (SCRI). Experiments were performed in compliance with the SCRI Animal Care and Use Committee.

#### LN studies

For the experiment shown in Figure 2, a male C57BL/6 mouse was adoptively transferred with 10^6 naive OT-II CD4+ T cells and one day later immunized in the footpad with OVA plus Alum; 4.5 days later the draining popliteal LN was harvested and used for analysis. For the experiments shown in Figure 5, non-draining steady-state skin LNs were harvested from male C57BL/6 mice which were previously injected in the contralateral distal footpad with Alum 2 days before harvest. For the experiments shown in Figures 6 and 7, skin LNs were harvested from naive male C57BL/6 mice. No differences in the localization or abundance of the various myeloid cell types under investigation have been observed between naive versus non-draining baseline LNs.

#### Tumor studies

Balb/C mice were injected subcutaneously with 5×10^6 CT26.WT cells and 9 days later, the tumor was harvested for fixation and imaging.

#### Mtb studies

All infections were done with a stock of Mtb H37Rv, as previously described (Urdahl et al., 2003). To perform aerosol infections, C57BL/6 mice were enclosed in a Glas-Col aerosol infection chamber, and Mtb bacilli were deposited directly into their lungs. Lungs were removed 34 days post infection.

### METHOD DETAILS

#### Tissue preparation – thin sections

All thin tissue sections were fixed with Cytofix (BD Biosciences) buffer diluted 1:3 with PBS for 12h at 4C and then dehydrated with 30% sucrose in PBS for 12–24h at 4C. Tissues were next embedded in O.C.T. compound (Tissue-Tek) and stored at 80C. LNs were sectioned on a Thermo Scientific Microm HM550 cryostat into 20 mm sections and were then prepared and imaged as previously described (Gerner et al., 2012). Briefly, sections were stained with panels of fluorescently conjugated antibodies, shown in Table S1, coverslipped with Fluoromount G mounting media (SouthernBiotech), and imaged on a Leica SP8 microscope. Antibody panels were designed to detect various innate and adaptive immune populations. In some analyses, limitations to the maximum number of analytes per panel precluded the discrimination of certain populations, such as migratory cDC1 and Langerhans cells. Certain discriminatory markers typically used in flow cytometry, such as cDC1-associated CD103 and XCR1, are not easily usable for confocal imaging due to epitope loss after paraformaldehyde fixation.

#### Tissue preparation – thick slices

Volumetric imaging using Ce3D tissue clearing (thick sections) was performed as previously described (Li et al., 2017, 2019). In brief, LNs were fixed with Cytofix (BD Biosciences) buffer diluted 1:3 with PBS for 12–20h at 4C. Excess fat was carefully removed using a dissection microscope, and the samples were embedded in 2% Agarose. 500 mm thick cross-sectional slices were generated using a Vibratome (Leica VT1000S, Speed: 215 Frequency: 8). Slices were next placed in blocking buffer (1%NMS, 1%BSA, 0.3%Triton, in 0.1MTris) for 24h at 24C on a rocker. After blocking, LN slices were stained with a panel of directly conjugated antibodies (Table S1) for 3 days at 34C on a shaker, then washed in blocking buffer for one day at 24C. Next, slices were placed in Ce3D clearing solution (13.75ml 40% [vol/vol diluted with PBS] N-methylacetylamide; 20 g Histodenz; 25uL Triton X-100; 125ul Thioglycerol) at 24C for at least 24h. Finally, slices were coverslipped using Ce3D as the mounting media and imaged on a Leica SP8 microscope.

#### Imaging

All samples were imaged using a Leica confocal SP8 microscope, with either a 40X 1.3NA (HC PL APO 40x/1.3 Oil CS2, for 20 mm sections) or a 20X 0.75NA (HC PL APO 20x/0.75 IMM CORR CS2, free working distance = 0.68 mm, for thick cleared slices) oil objective with type F immersion liquid (Leica, refractive index n_e_ = 1.5180). After acquisition, stitched images were compensated for spectral overlap between channels using the Leica Channel Dye Separation module in the Leica LASX software. For single stained controls, UltraComp beads (Affymetrix) were incubated with fluorescently conjugated antibodies, mounted on slides, and imaged with the same microscope settings used in the image they were being used to compensate. In all figures, for visual clarity, thresholds were applied to the displayed channel intensities.

### QUANTIFICATION AND STATISTICAL ANALYSIS

#### Image analysis and histo-cytometry

Image analysis and histo-cytometry was performed as described previously, with minor modifications (Gerner et al., 2012; Li et al., 2017, 2019). Briefly, using Imaris, various steps were taken to process the images, identify the signal that belongs to each individual cell (segment cell objects) or identify where a signal is without identifying specific cells that signal belongs to (generate landmark spots). Generating spots creates spherical objects centered on any pixels with signal above a user set threshold (Table S3). These spots allow for rapid digitization of the location of signal, without the need for accurate segmentation of cell bodies, and is useful for landmark channels, where the respective signal is not used to annotate specific cell types, but only to delineate the physical borders of specific tissue regions. Unless otherwise noted, only the channel arithmetic and surface or spot creation steps for each sample shown in Table S3 were used to process the images. Channel arithmetic were performed using either the default Imaris function or a customized ImarisXT extension, *Calebs_Multi_EQ_ChannelArithmetics_V3*, which allows for batching multiple equations and auto saving the image once the function is done running. Additional normalization and processing steps for specific samples are described below. After surface creation, the MFI for each imaged channel, as well as the volume, sphericity, and position of the cell objects were exported and concatenated into a single .csv file using the *Imaris_To_FlowJo_CSV_Converter_V4* MATLAB function, which is a customized .csv merge tool. The combined .csv file was next imported into FlowJo (TreeStar) and the cell objects were classified into the indicated cell subsets according to the gating strategies shown in the respective figures.

For the experiments described in Figure 2, using Imaris version 8.3 the All Cell Composite channel was created using the equation in Table S3. Using the surface creation tool in Imaris, masking surfaces were created around this new channel using the parameters for surface_1 shown in Table S3. Using the mask option, all pixels from the new channel outside the boundaries of this surface were set to equal 255 (the maximum pixel intensity). Next the new channel was smoothed with a Gaussian filter with a width of 0.56 mm. This new channel was then inverted, and the gamma was set to 0.3. Finally, cell object surfaces were created using the inverted new channel with the properties shown in Table S3 for surface_2.

For both experiments shown in Figure 6, all image layers were first normalized along the Z axis. For experiment 1, a clean CD31 channel was created to correct for residual channel spillover from the Lyve1 channel. Next a composite channel of myeloid cell markers was created according to the equation in Table S3. Finally, surfaces were created according to the parameters in Table S3. For the images from the second experiment, channels SIRPa, B220, MHC-II, CD31, and CD11c were smoothed using the Imaris Gaussian Smooth function with a width of 0.56 mm. Next, a composite channel of myeloid cell markers was created according to the equation in Table S3. Finally, surfaces were created according to the parameters in Table S3.

For the image of the lung shown in Figure 4, a composite of all surface markers was created using the equation in Table S3. The linear stretch function in Imaris was used to set the blackpoint of this channel to 40, gamma was adjusted to 0.75, and Gaussian smoothing was used with a width of 0.32um. The Jojo1 nuclear stain channel was brightened with the linear stretch function in Imaris to set the saturation point to 200, gamma was adjusted to 1.5, and Gaussian smoothing was used with a width of 0.32um. The all surface marker channel was then subtracted from the altered Jojo1 channel to form a “Nuclei” channel according to Table S3. Finally, surface objects were created in Imaris on this new channel using the parameters outlined in Table S3. A small number of the Mtb^+^ objects were identified outside the granuloma in the images. These appeared extra-cellular and likely represented imaging artifacts; they were not selectively removed from analysis to avoid introducing user bias.

#### Statistical analysis

No statistical method was used to predetermine sample size. The statistical significance of Pearson’s correlation was calculated using a Student’s t distribution for a transformation of the correlation.

#### CytoMAP spatial analysis

CytoMAP was written using MATLAB version 2018b (Mathworks). A detailed description of the workflow and functions built into CytoMAP is available in the online user manual. Below is a brief discussion of the analysis used for the datasets described in this manuscript. The annotated cell surfaces for each dataset were loaded into CytoMAP by importing the corresponding paired .wsp and .fcs files after saving them in FlowJo. These collectively contain the cell statistics, gate definitions, and spatial positions for each cell object. Once imported the following functions were used in CytoMAP to analyze the data.

#### Generate Random Points

This function, found in the ribbon at the top of the figure window, uses MATLAB’s built in rand function, with all default parameters, to generate a set of random points based on the set of currently plotted points in the *New Figure* window. This function only generates randomized data with respect to the axes which are plotted, while setting all other channel values to 0 for the new set of points. Additionally, this function scales the randomized values to be within the limits of the currently plotted points. To generate points with a random spatial distribution, all cells were first plotted with positions on the X and Y axes. Next, the Generate Random Points function was called and this newly generated set of points was saved. Finally, all points outside the spatial bounds of the tissue were discarded by gating on objects within the tissue boundaries, resulting in sets of RDPs for each sample analyzed, with a representative set of points shown in Figure S4B.

##### Make Surface

This function uses MATLAB’s alphaShape function, with user defined parameters, to wrap the currently plotted points in a surface. This surface can then be used to gate on points, such as randomly distributed points, and to calculate the distance to region borders. This function was used to generate the surfaces shown in Figure 5F.

##### Calculate Distance

This function finds the physical distance, either to the border of user defined surfaces, or to the nearest cell. This function was used to find the distance to region borders plotted in Figure 5 G–J, and the distance between myeloid cells and the nearest blood vessel plotted in Figure 6B.

##### Raster Scan Neighborhoods

This function uses Neighborhood analysis, which finds the local composition of cells within a circular (2D data) or spherical (3D data) area/volume in the tissue. If the data have non-zero z dimensionality but the z thickness is less than the radius of the neighborhoods, this function will treat the data as effectively 2D and use a cylindrical neighborhood window. This function calculates the number of cells and the MFI of each channel summed over all cells in each neighborhood. The positions of the neighborhoods are evenly distributed throughout the tissue in a grid pattern with a distance between neighborhood centers of half of the user defined radius. The neighborhood information can then be used for further analysis (e.g., local cellular densities, cell-cell associations). Unless otherwise noted, all neighborhoods described in this manuscript were defined using this function.

##### Cell Centered Neighborhoods

This function is similar to Raster Scan Neighborhoods, except the position of the neighborhoods are centered on a user selected cell type. Once the cell type is chosen and the radius is defined, this function calculates the number of cells and the MFI of each channel summed over all cells in each neighborhood. Only the neighborhoods described in Figure 7, and S7 were defined using this function. *Classify Neighborhoods Into Regions*

This function is used to define tissue regions using multiple types of information about the neighborhoods. This includes, the standardized number of cells of each phenotype in each neighborhood (number of cells minus mean number of cells, divided by the standard deviation of the number of cells in each neighborhood across the dataset), composition (number of cells divided by the total number of cells in each neighborhood) and raw number of cells per neighborhood, which were used as indicated in the respective figures. In this manuscript, the physical position of the neighborhoods was not used for region definition and the minimum of the Davies-Bouldin function was used to automatically determine the number of regions. The neighborhoods were clustered using the SOM function. This utilizes MATLAB’s selforgmap function with default parameters, except the dimensions options, which is equal to the number of regions (NR) by one, i.e.

app.net.(ModelName).Network=selforgmap([NR,1])

Once the network parameters are defined, the network is trained on the neighborhood data using MATLAB’s train function. This assigns a cluster number to each neighborhood. The selforgmap algorithm starts with NR “neurons” positioned throughout the data. It then iteratively moves the position of the neurons closer to the data to match the landscape of the data. Here, the position is not the spatial position, but the topological position within the cell composition data. The neighborhoods are then clustered by finding the closest neuron for each neighborhood. For visualization purposes the arbitrarily color designations of the individual regions were changed using the Edit Region Colors function in CytoMAP. The composition of the color-coded neighborhoods was plotted using the Heatmap Visualization function in CytoMAP. The spatial distribution of the regions was visualized by generating a new figure in CytoMAP, plotting the positions of the neighborhoods, and selecting the regions for the ‘c’ axis to color-code the neighborhoods by region type.

##### Reduce Dimensions

Dimensionality reduction algorithms can be used to visualize tissue structure and complexity, as well as for sample-to-sample comparison. These dimensionality reduction techniques also help reveal how the tissue neighborhoods are organized to generate global tissue structure. In CytoMAP this function uses MATLAB implementations of multiple dimensionality reduction techniques. Information about the neighborhoods including the standardized number of cells of each phenotype in each neighborhood (number of cells minus mean number of cells, divided by the standard deviation of the number of cells in each neighborhood across the dataset), composition (number of cells divided by the total number of cells in each neighborhood) or raw number of cells per neighborhood can be used. For t-SNE and PCA we used the built in MATLAB implementations. For the t-SNE plots in this manuscript the default MATLAB options were used, including: Euclidian distance, perplexity of 30, theta, of 0.5, and exaggeration of 4. We used the e4 MATLAB implementation of UMAP, with default parameters, provided by the Herzenberg Lab at Stanford University available for download at: https://www.mathworks.com/matlabcentral/fileexchange/71902-uniform-manifold-approximation-and-projection-umap.> We used the MATLAB implementation of PHATE, with default parameters, provided by Krishnaswamy Lab available for download at: https://github.com/KrishnaswamyLab/PHATE.

##### Pseudo-Space

Pseudo-space, reduces the complexity of cell distribution across tissues into a one-dimensional plot, helping reveal the fundamental positional relationships of cells with respect to one another. This function allows the user to sort the neighborhoods by the absolute number or composition of different cell types within the neighborhoods and plots the neighborhoods in this sorted order on a linear Pseudo-space axis. The y axis is normalized to allow comparison between cell types, and the data are smoothed along the x axis by a user defined amount. This function was used with the data type, weights, and smoothing parameters for each figure shown in Table S4.

##### Cell-Cell Correlation Analysis

The local cell density within individual neighborhoods can be used to correlate the location of different cell types, revealing which cell populations preferentially associate with one another, or conversely avoid one another. This function calculates the Pearson correlation coefficient of the number of cell or object types within the scanned neighborhoods and graphs these on a heatmap plot. This correlation analysis can be performed across multiple samples, and can be done either over entire tissues or within specified tissue regions. This is important, as cells may have distinct associations with one another in different tissue compartments.

##### Network Map

Region interaction network mapping calculates which regions preferentially border one another within the samples. This function calculates the percentage of neighborhoods of each region type which are directly in contact with each other region type. It creates a force directed graph with a node for each region type, where the nodes are connected by an edge if more than 0.005% of the neighborhoods of that nodes type are in contact with the connecting node’s region type. The edge thickness is proportional to the % of neighborhoods in contact with the connecting node region type, and the node size is proportional to the number of neighborhoods of the region type.

### DATA AND CODE AVAILABILITY

All data are available upon request. Imaris extensions and other scripts used for histo-cytometry analysis are available for download at: https://gitlab.com/gernerlab/imarisxt_histocytometry. CytoMAP software is available for download at: https://gitlab.com/gernerlab/cytomap.

## Supplementary Material

1

2

3

4

## Figures and Tables

**KEY RESOURCES TABLE:** 

REAGENT or RESOURCE	SOURCE	IDENTIFIER
Antibodies		
All antibodies are listed in Table S5	N/A	N/A
Chemicals, Peptides, and Recombinant Proteins		
Tissue-Tek O.C.T. Compound	Electron Microscopy Sciences	Cat# 62550-01
BD Cytofix fixation buffer	BD Biosciences	Cat# 554655
PBS (pH 7.4)	Caisson Labs	Cat# PBL06-6X500ML
Triton X-100	Sigma-Aldrich	Cat# T-9284
Bovine Serum Albumin	Sigma-Aldrich	Cat# A9576-50ML
Normal Mouse Serum	Jackson Laboratories	Cat# 015-000-120
Tris buffer (1 M dilute to 0.1M)	Fisher Scientific	BP1756500
Mix-n-Stain CF Dye Antibody Labeling Kits	Biotium	Cat# 92433-92339
N-methylacetamide	Sigma-Aldrich	Cat# M26305
Histodenz	Sigma-Aldrich	Cat# D2158
1-Thioglycerol	Sigma-Aldrich	Cat# M1753
High-vacuum grease	VWR	Cat# DOWC1597418
Agarose	Fisher Scientific	Cat# 16500500
Immersion Oil, type F	Fisher Scientific	Cat# NC0586121
EndoFit Ovalbumin 100mg	Invivogen	Cat# vac-nova-100
Alhydrogel adjuvant 2%, 250mL	Invivogen	Cat# vac-alu-250
Sucrose, ultrapure DNase- and RNase-free	VWR	Cat# 97061-432
Experimental Models: Organisms/Strains		
Mouse: CD45.2 (B6) 4get OT-II	This paper	N/A
Mouse: B6.SJL (CD 45.1)	Jackson Laboratories	Strain # 002014
Mouse: C57BL/6j	Jackson Laboratories	Strain # 000664
Mouse: Balb/C	Charles River	Strain # 028
Software and Algorithms		
CytoMAP	This Manuscript	https://gitlab.com/gernerlab/cytomap
Imaris extensions	This Manuscript	https://gitlab.com/gernerlab/imarisxt_histocytometry
Imaris	Bitplane	https://imaris.oxinst.com/
LASX	Leica Microsystems	https://www.leica-microsystems.com/products/microscope-software/p/leica-las-x-ls/
FlowJo	FlowJo, LLC	https://www.flowjo.com/
Prism	GraphPad Software	https://www.graphpad.com/scientific-software/prism/
MATLAB	The MathWorks, Inc.	https://www.mathworks.com/products/matlab.html?s_tid=hp_products_matlab
Other		
UltraComp eBeads Compensation Beads	Fisher Scientific	Cat # 01-2222-42
PAP pen	Vector Laboratories	Cat# H-4000

## Highlights

- CytoMAP is a user-friendly, comprehensive platform for spatial analysis of tissues
- Allows quantification of cellular positioning and global tissue structure
- Enables exploration of cellular and tissue microenvironment heterogeneity
- CytoMAP reveals fundamental features of myeloid cell organization in lymph nodes
