# Supplementary material for: CytoMAP: A Spatial Analysis Toolbox Reveals Features of Myeloid Cell Organization in Lymphoid Tissues
Source: Cell Rep. Author manuscript; Available in PMC 2020 May 18. (PMC7233132; doi:10.1016/j.celrep.2020.107523)
Supplement: 1 [file NIHMS1586767-supplement-1.pdf]

Cell Reports, Volume 31

Supplemental Information

CytoMAP: A Spatial Analysis Toolbox Reveals Features of Myeloid Cell Organization in Lymphoid Tissues

Caleb R. Stoltzfus, Jakub Filipek, Benjamin H. Gern, Brandy E. Olin, Joseph M. Leal, Yajun Wu, Miranda R. Lyons-Cohen, Jessica Y. Huang, Clarissa L. Paz-Stoltzfus, Courtney R. Plumlee, Thomas Pöschinger, Kevin B. Urdahl, Mario Perro, and Michael Y. Gerner

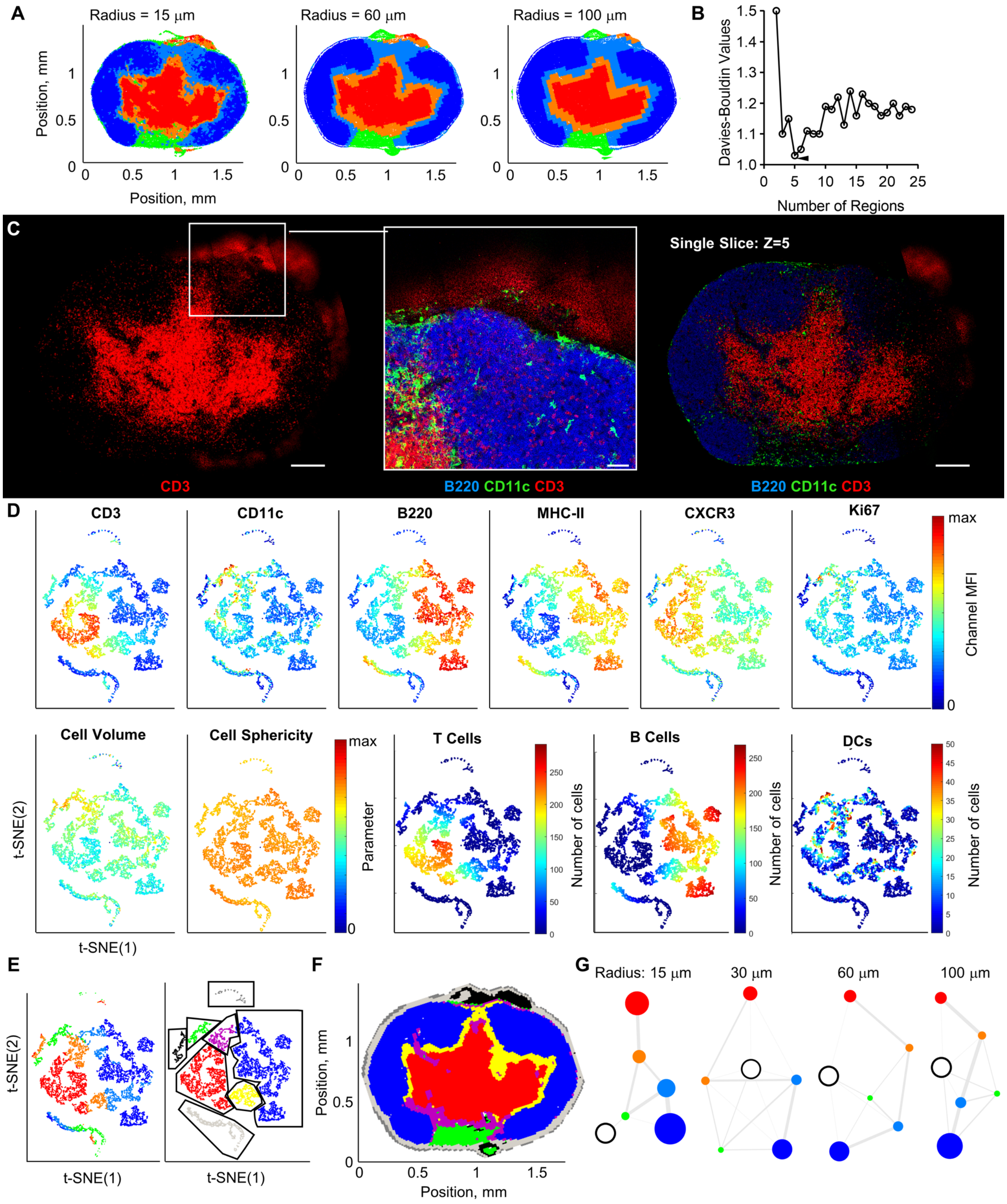

Figure S1. Identification of basic LN architecture with CytoMAP, Related to Fig. 2. A, Positional plots of neighborhoods obtained by using the indicated radius for raster scanning, with the neighborhoods being color coded by regions obtained from SOM clustering. **B**, The minimum Davies-Bouldin value (denoted by the arrow) was used to determine the number of regions presented in Fig. 2D. **C**, The same LN dataset as presented in Fig. 2A, demonstrating the out-of-plane noise in the CD3 channel within the maximum

projection image (left) or a single Z-slice (Z=5, right). Overview image scale bar = 200µm; zoom-in scale bar = 50µm. **D**, Extended t-SNE analyses, for t-SNE plots made using the non-standardized composition of the neighborhoods. These plots are color-coded heatmaps of the total MFI signal or number of cells in each neighborhood for the indicated markers or cell types. **E**, Same t-SNE as in panel D, with the neighborhoods color-coded according to the regions defined in Fig. 2D on the left and manually drawn gates on the right. **F**, Gated neighborhoods were positionally remapped using the color definitions from the gates shown in the right plot in panel E. **G**, Interaction map showing the percentage of shared border between regions, defined using varying neighborhood radii, also shown in panel B. The regions found with a radius of 30µm are from Fig. 2D. The white node represents area external to the tissue. The node size is proportional to the prevalence of that region within the sample (number of neighborhoods per image). Lines connect regions that share borders, with the line thickness being proportional to the percent of the border shared between the regions.

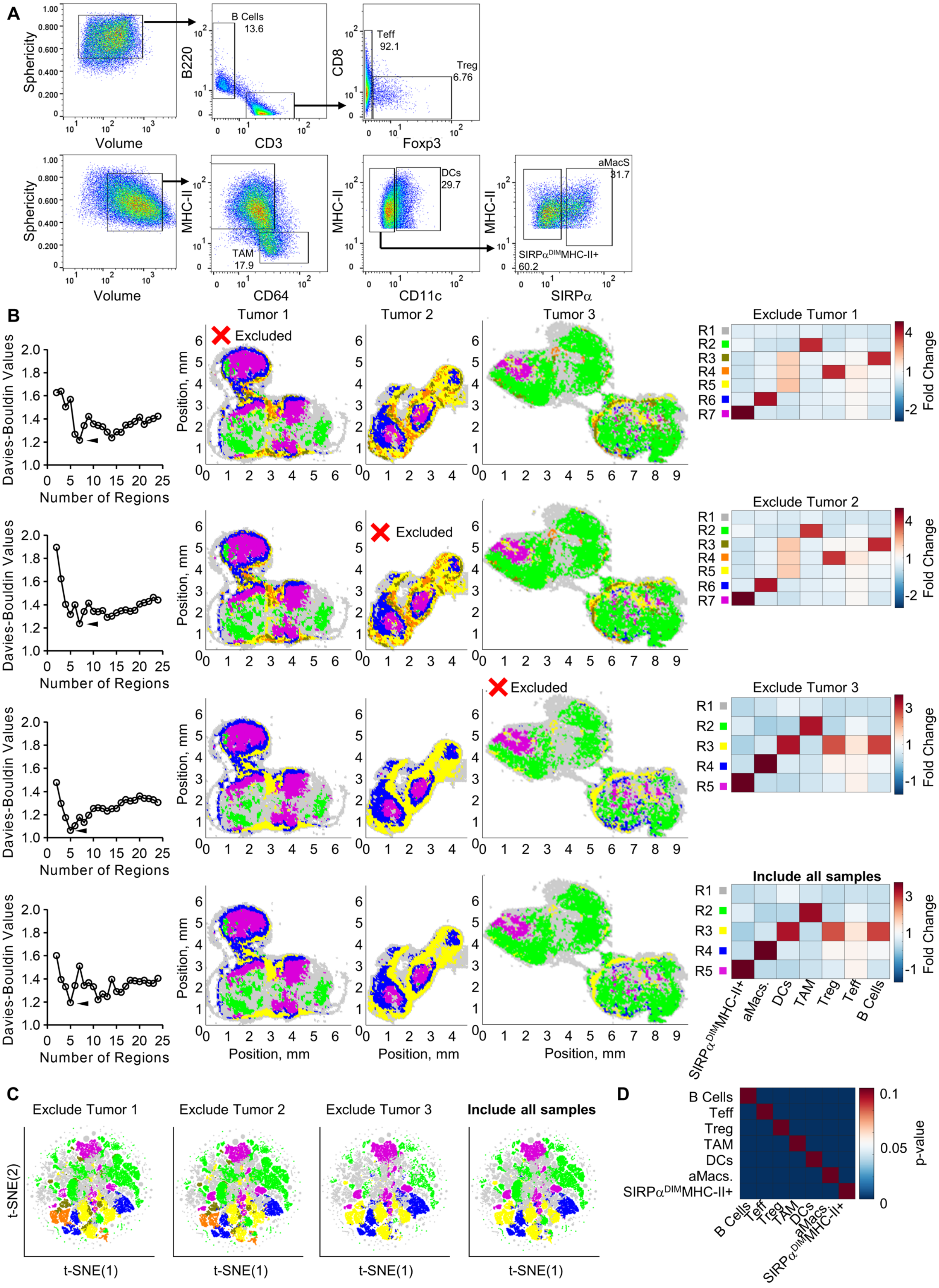
A
Sphericity
Volume
B220
CD3
B Cells 13.6
CD8
Foxp3
Teff 92.1
Treg 6.76
MHC-II
CD64
TAM 17.9
CD11c
DCs 29.7
SIRPα
aMacS 31.7
SIRPα^DIM MHC-II+ 60.2
B
Tumor 1
Tumor 2
Tumor 3
Excluded
Davies-Bouldin Values
Number of Regions
Position, mm
Exclude Tumor 1
Exclude Tumor 2
Exclude Tumor 3
Include all samples
Fold Change
R1
R2
R3
R4
R5
R6
R7
SIRPα^DIM MHC-II+
aMacs.
DCs
TAM
Treg
Teff
B Cells
C
Exclude Tumor 1
Exclude Tumor 2
Exclude Tumor 3
Include all samples
t-SNE(1)
t-SNE(2)
D
B Cells
Teff
Treg
TAM
DCs
aMacs.
SIRPα^DIM MHC-II+
p-value

Figure S2. Quantitative analysis of tumor immune infiltrate, Related to Fig. 3. A, Histo-cytometry plots of the gating strategy used to annotate the cell types for the sample analyzed and presented in Fig. 3. **B**, Comparison of regions between four models trained on either all samples (bottom row of panel, also presented in Fig. 3C-F), or all samples except the sample denoted with the red X. On the left column of this panel are the Davies-Bouldin values, with the minimum denoted by the arrow. The middle three columns show the spatially remapped color-coded neighborhoods. The rightmost column of this panel shows the normalized fold change (fold change in the number of cells per neighborhood from each region, compared to the average number of cells in neighborhoods from all regions and samples). **C**, t-SNE plots of neighborhoods, color coded according to the corresponding model, as defined in panel B. **D**, Heatmap of the p-values for the corresponding Pearson correlation coefficients presented in Fig. 3H. High p-values correspond to correlation coefficients not significantly different from 0.

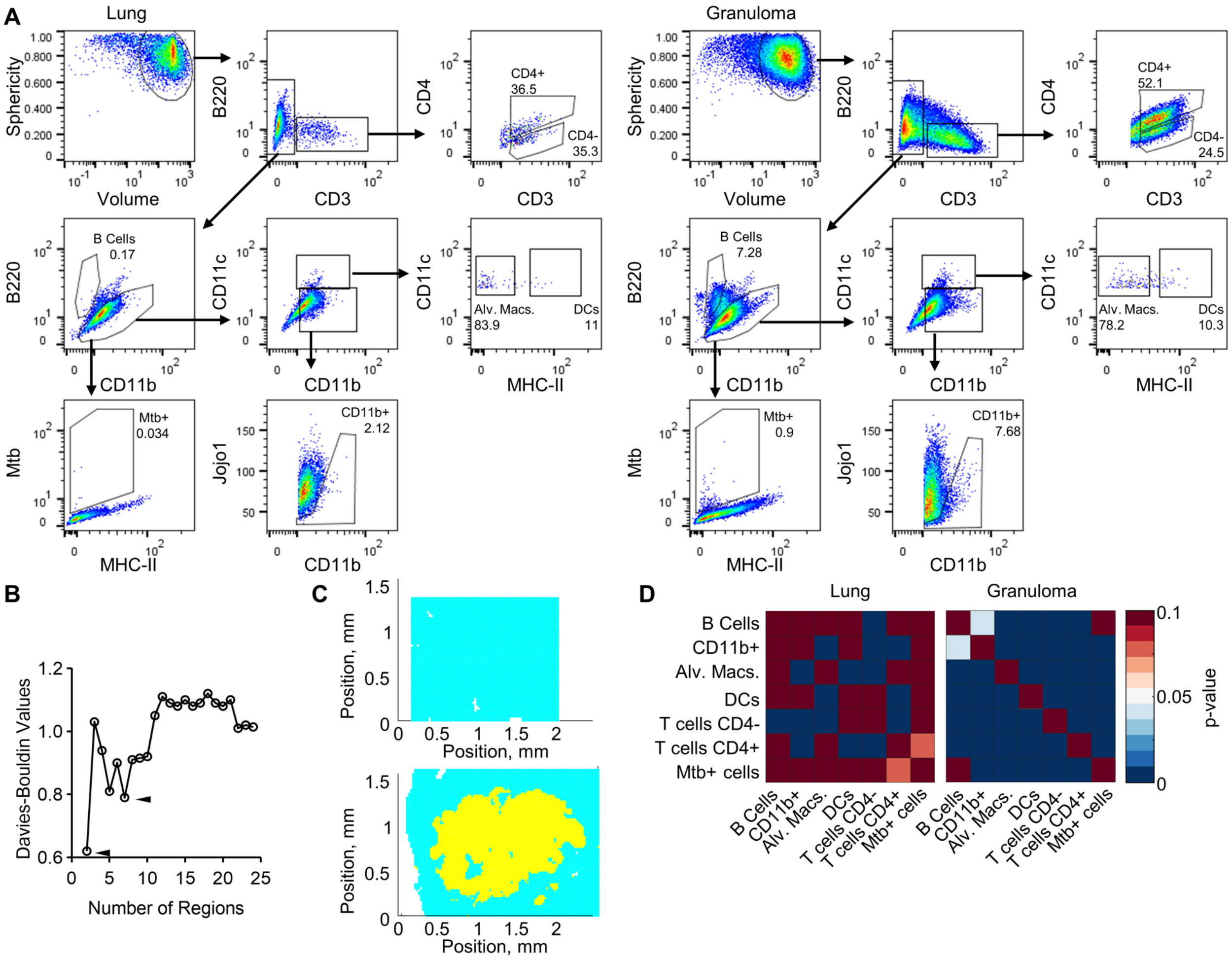

Figure S3. Quantitative analysis of lung Mtb granulomas, Related to Fig. 4. A, Histo-cytometry plots showing the gating strategy used to annotate the cell types presented in Fig. 4B. The cell objects extracted from the uninvolved lung image are on the left and the granuloma image are on the right. **B**, The Davies-Bouldin values used to determine the number of regions in the neighborhood dataset, with the SOM clustering presented Fig. 4C. The neighborhoods were clustered into 6 regions based on these values. Since there are two very different regions in this dataset (lung and granuloma) the minimum of the Davies-Bouldin yields only two regions. However, the local minimum at 6 regions reveals both the difference between the lung and granuloma neighborhoods, as well as the different regions within the granuloma. **C**, The color-coded position plot of the regions if 2 regions were used based on the absolute minimum from the Davies-Bouldin function. This plot demonstrates two distinct regions, the uninvolved lung (cyan) and the granuloma (yellow). **D**, The p-values for the corresponding Pearson correlation coefficients presented in Fig. 4F. High p-values correspond to correlation coefficients not significantly different from 0.

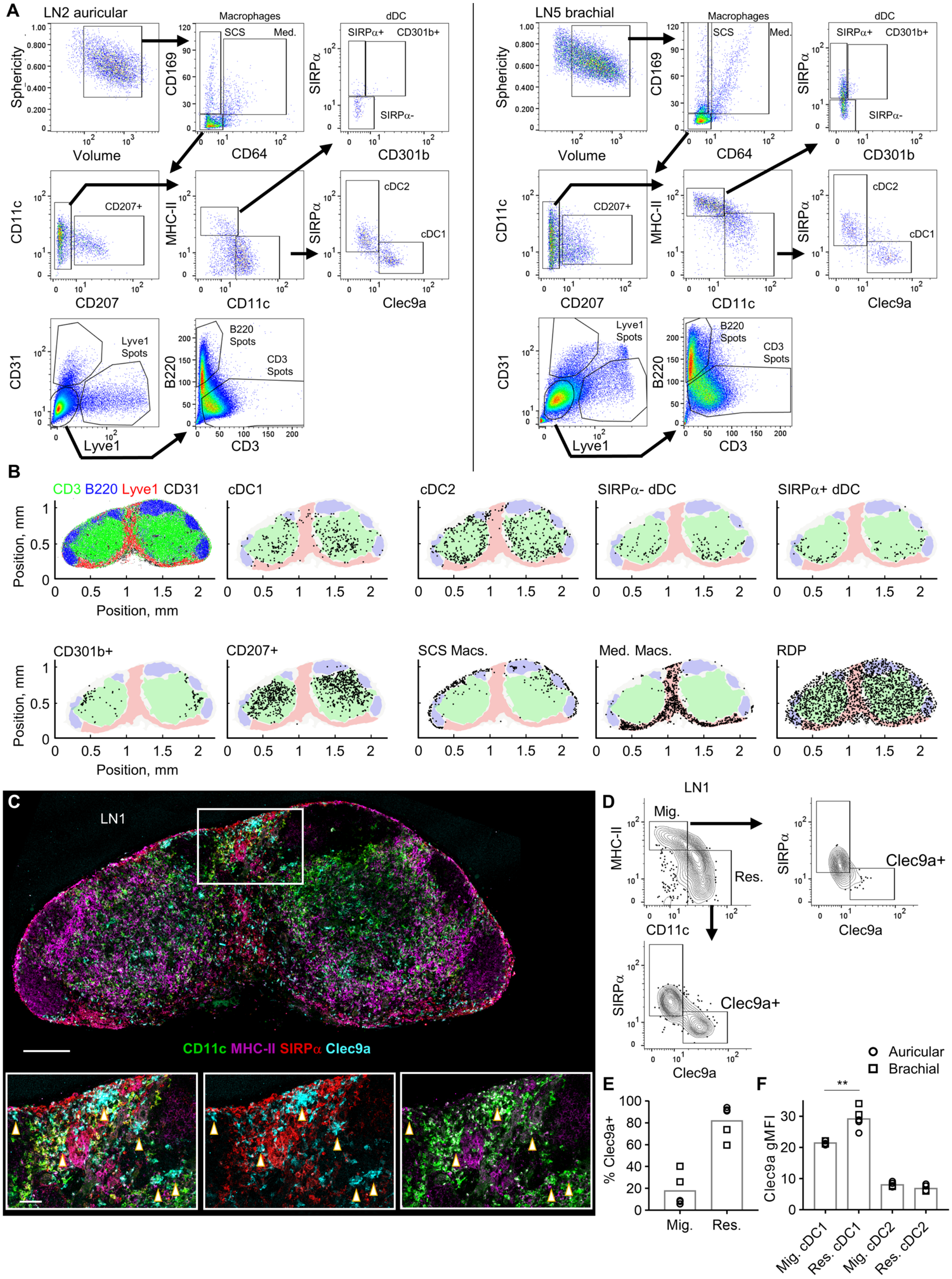
A
LN2 auricular
LN5 brachial
Sphericity
Volume
Macrophages
SCS
Med.
CD169
CD64
dDC
SIRPα+
CD301b+
SIRPα-
SIRPα
CD301b
CD11c
CD207
CD207+
MHC-II
cDC2
cDC1
Clec9a
CD31
Lyve1
Lyve1 Spots
B220
B220 Spots
CD3 Spots
CD3
B
CD3 B220 Lyve1 CD31
cDC1
cDC2
SIRPα- dDC
SIRPα+ dDC
CD301b+
CD207+
SCS Macs.
Med. Macs.
RDP
Position, mm
C
LN1
CD11c MHC-II SIRPα Clec9a
D
LN1
Mig.
Res.
Clec9a+
E
% Clec9a+
Mig.
Res.
F
Auricular
Brachial
Clec9a gMFI
Mig. cDC1
Res. cDC1
Mig. cDC2
Res. cDC2
**

Figure S4. Quantifying myeloid cell organization in LNs, Related to Fig. 5. A, Two independent representative histo-cytometry plots demonstrating the gating strategy used to annotate cell types and landmark spots in the dataset presented in Fig. 5. **B**, Positional plots of the landmark spots (top left), indicated cell populations, and RDP (bottom right) overlaid on the manually annotated regions, as shown in Fig. 5F. **C**, Multi-parameter confocal microscopy image of the same representative LN shown in Fig. 5A. Scale bar = 200µm, zoom-in scale bar = 50µm. Arrows on zoom-in denote Clec9a+ cells. **D**, Histo-cytometry gating showing increased expression of Clec9a on resident cDC1 cells. **E**, Bar graph showing the percentage of Clec9a+ cells that are in the migratory or resident DC gates. **F**, Bar graph showing the mean Clec9a expression of Clec9a+ migratory and resident cDCs.

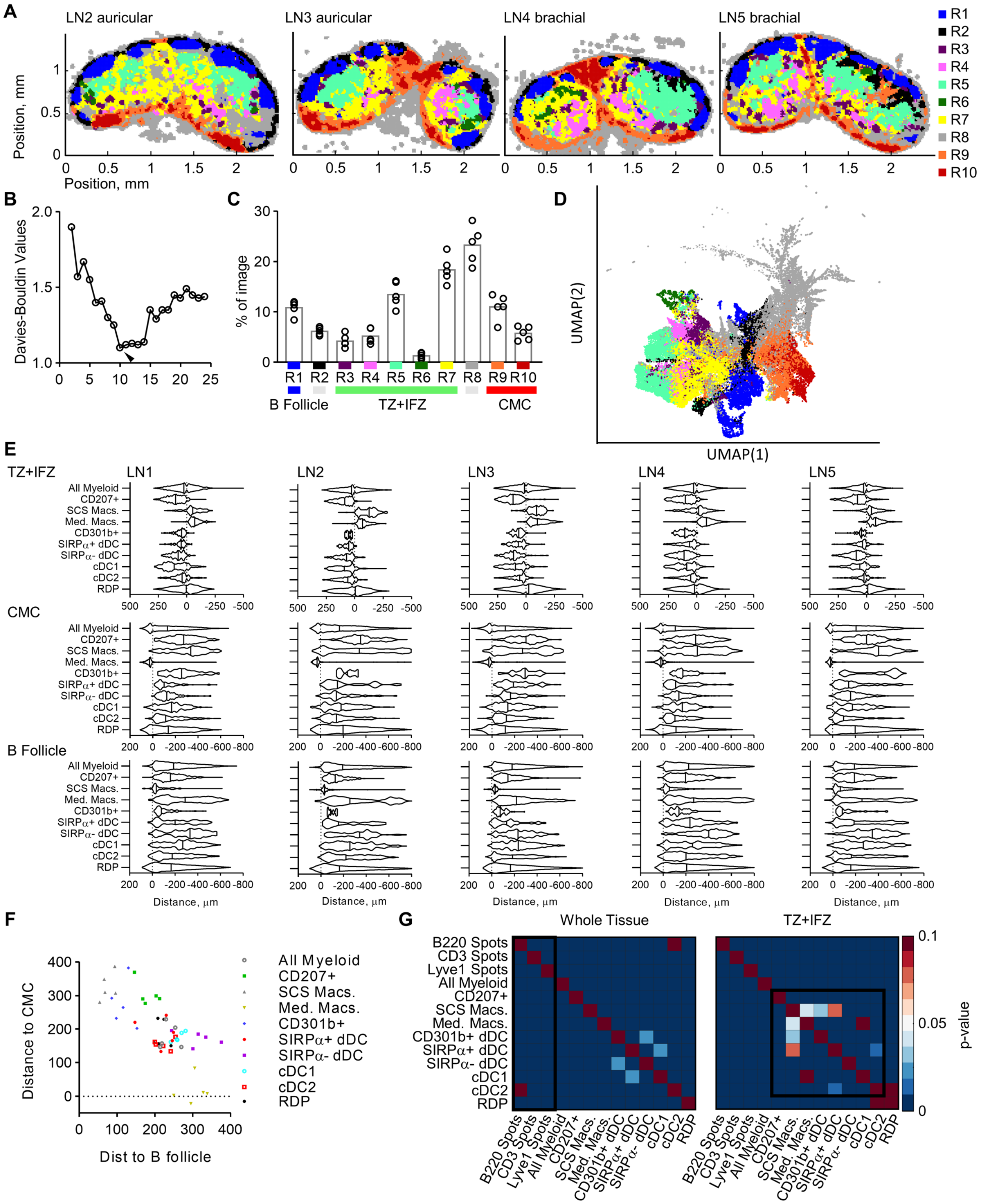

Figure S5. CytoMAP reveals patterns of myeloid cell organization in LNs, Related to Fig. 5. A, Color-coded spatial plots of the neighborhoods, demonstrating the distribution of CytoMAP generated regions across all of the LNs presented in Fig. 5. **B**, The minimum Davies-Bouldin value, denoted by the arrow, used to determine the number of regions presented in Fig. 5D. **C**, Region prevalence plot showing the percentage of the neighborhoods from each sample in each region. Manually annotated region groups, used

to define the surfaces shown in Fig. 5F, are shown in the bottom color label. **D**, UMAP plot of neighborhoods from all samples, as color coded by region type shown in panel A. **E**, Violin plots of the distances for all cells from each LN. The means of these distances are shown in Fig. 5H-J. TZ+IFZ distance analysis for LN1 are the same data as presented in Fig. 5G. **F**, Combined analysis for Fig. 5I and 5J. Plot demonstrates the average distance of the indicated cell populations to the B cell follicle versus the CMC, with each dot representing an individual sample. **G**, Heatmaps of the p-values for the Pearson correlation coefficients shown in Fig. 5K. High p-values correspond to correlation coefficients not significantly different from 0. Data represent 5 samples from one experiment.

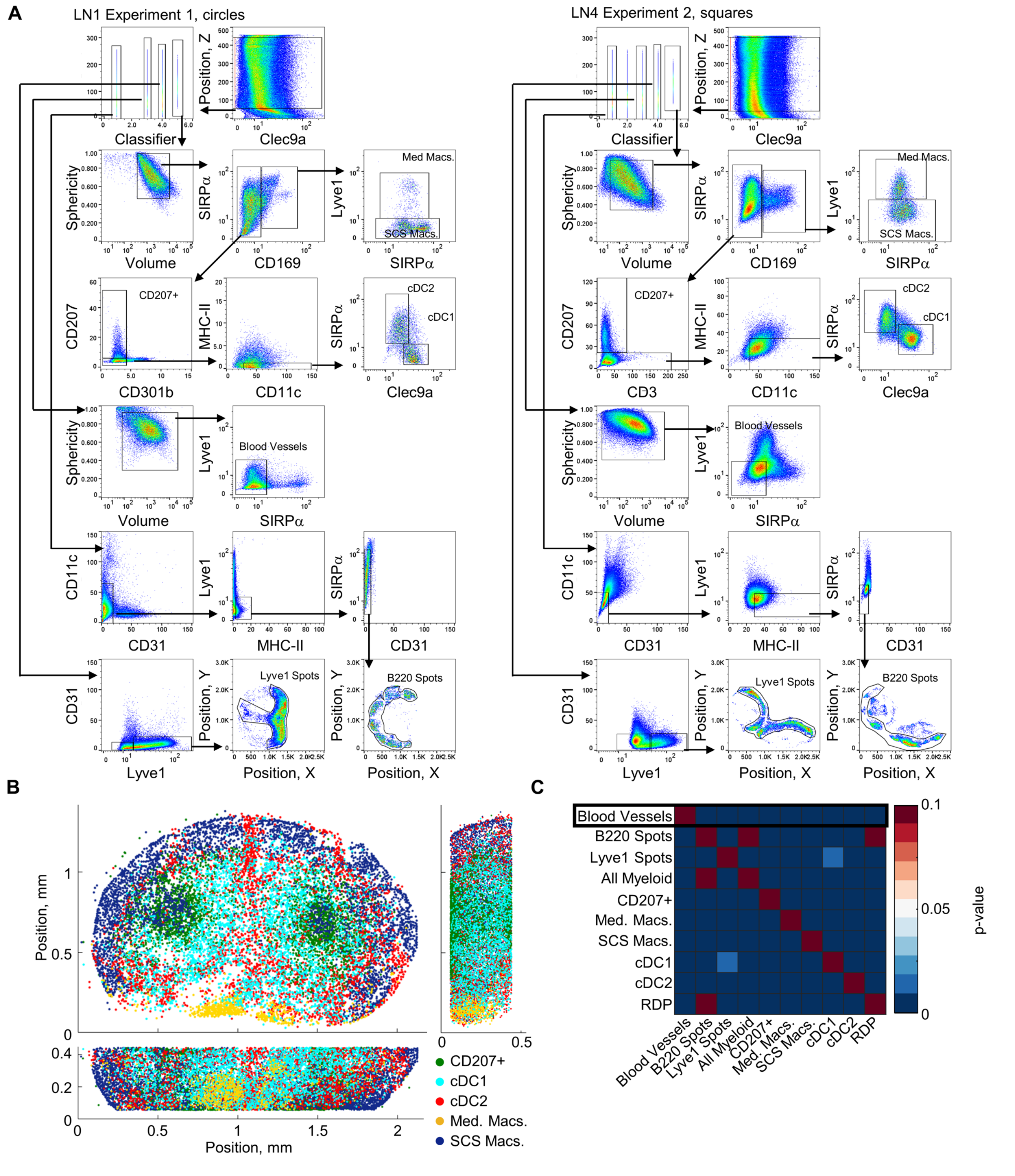

Figure S6. Identification of DC subsets in 3D LNs, Related to Fig. 6. A, Histo-cytometry hierarchical gating of the cell populations extracted from two representative samples, one for each experiment, for the data presented in Fig. 6. The classifier is a manually added parameter used to distinguish between different sets of surface objects for gating purposes. **B**, 3D spatial remapping of the cell objects annotated in panel A. **C**, Heatmap of the p-values for the Pearson correlation coefficients presented in Fig. 6E. High p-values correspond to correlation coefficients not significantly different from 0. Data represent four samples from two independent experiments.

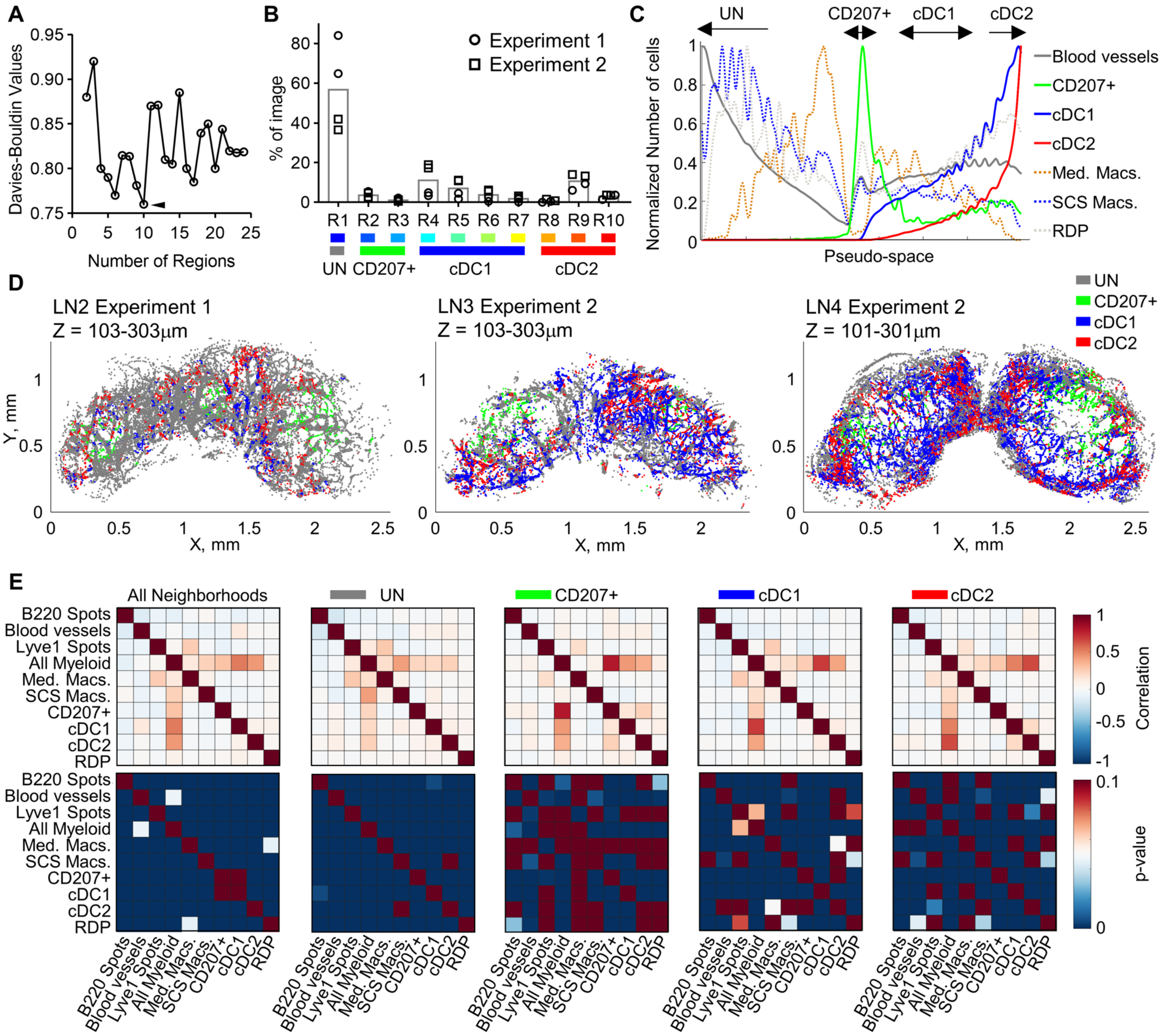

Figure S7. Characterization of blood vessel branches in LNs, Related to Fig. 7. A, The minimum Davies-Bouldin value, denoted by the arrow, was used to determine the number of regions used for SOM clustering presented in Fig. 7C. **B**, Percentage of neighborhoods in each cluster as defined in Fig. 7C. **C**, Pseudo-space plot of the neighborhoods from all 4 samples showing the cellular relationships across the different types of vascular neighborhoods. **D**, Positional plots of the vascular neighborhoods in 200µm thick virtual Z sections, also color-coded based on the manual annotations in the bottom color bar of Fig. 7C and panel B. **E**, Heatmap showing the Pearson correlation coefficients between the number of different cell or landmark object types per neighborhood for neighborhoods from all samples. Correlation was either calculated for all neighborhoods from all regions, or only neighborhoods from a specific region. Data represent four samples from two independent experiments.

Table S1. Antibody staining panels, Related to STAR Methods

Ch	Antibody	Fluorophore
**Fig. 2**		
1	CD3	BV421
2	TCF1	PacBlue
3	CD45.2 (OT-II)	BV510
4	CXCR3	PE
5	MHC-II	AF594
6	Ki67	AF700
7	B220	CF750
8	anti-GFP	AF488
9	CD11c	AF647
**Fig. 5**		
1	CD64	PE
2	SIRPα	CF594
3	CD207	AF488
4	CD169	CF514
5	Lyve1-biotin	SA-490LS
6	CD11c	BV421
7	CD31	BV480
8	MHC-II	Dy395xl
9	Clec9a	CF633
10	CD301b	CF660
11	B220	AF700
12	CD3	APC-Fire750
**Fig. 6 Experiment #1**		
1	CD31	BV421
2	CD11c	BV480
3	B220	Dy405ls
4	Clec9a	CF633
5	CD207	AF488
6	CD169	CF514
7	SIRPα	CF594
8	Lyve1	eFluor570
9	CD301b	CF660
10	MHC-II	AF700
**Fig. 6 Experiment #2**		
1	CD169	CF514
2	CD3	AF700
3	Lyve1-biotin	SA-CF750
4	SIRPα	BV421
5	B220	BV510
6	MHC-II	Dy395xl
7	Clec9a	CF633
8	CD31	AF488
9	CD207	AF546
10	CD11c	eFluor615
**Fig. 3**		
1	SIRPα	BV421
2	CD11c	BV480
3	CD8	BV510
4	MHC-II	Dy396xl
5	Clec9a	αsheep-CF633
6	PCREB	AF488
7	IRF4	CF514
8	B220-biotin	SA-490ls
9	CD64	CF660
10	CD3	AF700
11	PD1	αgoat-CF750
12	Foxp3	eF570
13	PD-L1	CF594

Fig. 4		
1	iNOS	AF405
2	CD11c	BV480
3	CD11b	BV510
4	B220	DY405LS
5	CD3	CF633
6	pS6	AF488
7	IFNg	PE
8	Jojo-1 iodide	
9	IRF4	CF594
10	CD4	CF660
11	MHC-II	AF700
12	Mtb	α-rabbit-CF750

Table S2. Processing time for key CytoMAP analysis steps on a Laptop (8 GB RAM, Intel® Core™ i7-7500U CPU @ 2.70GHz 2.90GHz) vs. Workstation (192 GB RAM, Intel® Xeon® Gold 5122 CPU @3.60 GHz 3.60GHz (2 processors)) , Related to STAR Methods

Dataset	Fig. 2	Fig. 3	Fig. 4	Fig. 5	Fig. 7
Description	1 LN cross section	3 tumor cross sections	2 regions from lung cross section	5 LN cross sections	4 thick LN volumes
Total imaged volume, mm^3	0.03	1.8	0.12	0.115	3.72
Number of channels	9	13	12	12	10
Number of objects	139,399	171,155	176,647	454,790	867,420
Neighborhood radius, µm	30	50	50	30	20
Number of Neighborhoods	11,328	192,785	12,075	63,540	144,196
**Time to define neighborhoods, minutes**					
Laptop	2.5	18.8	3.2	17.7	203.1
Workstation	0.4	3.7	0.3	1.8	173.3
**Time to determine number of regions, minutes**					
Laptop	1.3	8.8	0.8	5	15.1
Workstation	1.1	6.2	0.7	3.5	9.8
**Time to cluster neighborhoods, minutes**					
Laptop	0.1	0.3	0.1	0.3	91.5
Workstation	0.1	0.2	0.1	0.3	8.7

Table S3. Image Analysis and histo-cytometry, Related to STAR Methods

**Channel Arithmetic**		
Channel name/Sample	Description	Equation
**Fi. 2**		
All Cell Composite	CD3 + 0.5*CD45.2 + MHC-II	(ch1 .* 0.5) + (ch3.* 0.5) + ch5
**Fig. 3**		
Composite Lymphocyte	CD3+2*B220	ch10+(2.*ch8)
Composite Myeloid	CD11c+MHC-II+1.3*CD64	ch2+ch4+(1.3.*ch9)
**Fig. 4**		
Composite Surface Markers	[(CD11c>14)/175 + (CD11b>10.5)/160 + (B220>15)/130 + (CD3>30)/170 + (MHC-II>20)/165]*150	(ch2.*(ch2>14)./175 + ch3.*(ch3>10.5)./160 + ch4.*(ch4>15)./130 + ch5.*(ch5>30)./170 + ch11.*(ch11>20)./165).*150
Nuclei	Jojo1 – Composite Surface Markers	ch8 - ch13
**Fig. 5**		
clean-CD31	corrected spillover from CD11c	(ch7-ch6).*(ch7>ch6)
clean-SIRPα	corrected spillover from CD31	(ch2-ch7).*(ch2>ch7)
clean-CD169	exclude CD207+ subtract CD31	(ch4-ch13).*(ch4>ch13).*(ch3<70)
clean-CD11c	corrected spillover from CD31	(ch6-ch13).*(ch6>ch13)
clean-MHC-II	exclude B220	(ch8-ch11).*(ch8>ch11)
Composite Myeloid	clean-SIRPα + CD207 + clean-CD169 + clean-CD11c + clean-MHC-II + Clec9a	(ch14.*0.75) + (ch3.*0.75) + (ch15.*0.5) + (ch16.*0.9) + (ch17.*0.75) + (ch9 .* 0.75 .*(ch9>50))
**Fig. 6 Experiment #1**		
Clean CD31	CD31-0.1*Lyve1	(ch1-0.1.*ch8).*(ch1>0.1.*ch8)
Composite Myeloid	CD11c + Clec9a + CD207 + CD169 + SIRPα + CD301b + MHC2	ch2+ch4+ch5+ch6+ch7+ch9+(ch10.*4)
**Fig. 6 Experiment #2**		

Composite Myeloid	CD169 + 2*SIRPα + 2.5*MHC-II(B220<55) + Clec9a+CD207 + 1.5*CD11c - B220 - CD3 + min("")	((1.5.*ch1.*(ch1>5) + 3.8.*ch4.*(ch4>5) + 2.5.*ch6.*(ch6>5).*(ch5<40) +2.*ch7.*(ch7>5) + ch9.*(ch9>5) + 2.*ch10.*(ch10>5)-0.5.*ch3-0.5.*ch5) +min(min((1.5.*ch1.*(ch1>5) + 3.8.*ch4.*(ch4>5) + 2.5.*ch6.*(ch6>5).*(ch5<40) +2.*ch7.*(ch7>5) + ch9.*(ch9>5) + 2.*ch10.*(ch10>5)-0.5.*ch3-0.5.*ch5)))./2

Surface creation parameters

Surface Spots Name	Source Channel	Smoothing: Surface Detail (μm)	Background Subtraction: Diameter of Largest sphere	Absolute Intensity Threshold	Split Touching Objects: Split seed diameter (μm)	Quality Threshold	Voxel Number Threshold	Sphericity Threshold	Diameter (Spots)
**Fig. 2**									
Surface_1	All Cell Composite	20		8					
Surface_2	Inverted Ch	0.655		107	3.28	8.28	10-2781	0.583	
**Fig. 3**									
Myeloid Surfaces	Composite Myeloid	0.6	11	20	11	5	50-7103		
Lymphocyte Surfaces	Composite Lymphocyte	0.6	10	9	6	2.5	50		
**Fig. 4**									
All Cells	Nuclei	0.63	10	18.5	3.16	6.08	10		
**Fig. 5**									
Myeloid Surfaces	Composite Myeloid	0.758	25	7-22 (Sample Dependent)	7.5-10 (Sample Dependent)	4.5	300		
All Spots	B220					13.1			3
**Fig. 6 Experiment #1**									
Myeloid Surfaces	Composite Myeloid	1.5	12	20	12	10	50		
CD31 Surfaces	Clean CD31	2.5	15	2.25	4	3.5	150		
Lyve1 Spots	Lyve1		true			2			5
B220 Spots	B220		true LN1 (false LN2)			1.5 LN1 (7.5 LN2)			5
**Fig. 6 Experiment #2**									
Myeloid Surfaces	Composite Myeloid	0.8	11	40	10	6	50		
CD31 Surfaces	CD31	2	5	2	4	2.5	50		
Lyve1 Spots	Lyve1		false			20			5
B220 Spots	B220		false			40			5

Table S4. Pseudo-space input parameters, Related to STAR Methods

Fig. 2F<br>Data Preparation: Neighborhood Composition		
Cell Type	Weight	Smoothing
B cells	-1	500
T Cells	1	500
DCs	0.2	500
Fig. 3G<br>Data Preparation: Number of cells per Neighborhood		
B cells	1	10000
Teff	0	10000
Treg	5	10000
TAM	-2	10000
DCs	0	10000
aMACs.	1	10000
SIRPα^{DIM}MHC-II+	-1	10000
Fig. 4E<br>Data Preparation: Number of cells per Neighborhood		
B Cells	1	1000
Alv. Macs	-50	1000
CD11b+	0	500
DCs	0	1000
Mtb+ Cells	100	100

T Cells CD4-	1	1000
T Cells CD4+	1	1000
Fig. S7C<br>Data Preparation: Number of cells per Neighborhood		
Blood vessels	-0.1	5000
CD207+	1	5000
cDC1	10	5000
cDC2	20	5000
Med. Macs.	-0.1	5000
SCS Macs.	-0.1	5000
RDP	0	5000

Table S5. Antibodies, Related to STAR Methods

B220 -biotin (clone RA3-6B2)	BioLegend	Cat# 103204
B220 -BV510 (clone RA3-6B2)	BioLegend	Cat# 103247
B220 -AF700 (clone RA3-6B2)	BioLegend	Cat# 103232
B220 -CF750 (clone RA3-6B2) [Conjugated in house]	BioLegend	Cat# 103202
B220 -DY405LS (clone RA3-6B2)	Novus Biologicals	Discontinued
CD3 -BV421 (clone 17A2)	BioLegend	Cat# 100228
CD3 -AF700 (clone 17A2)	BioLegend	Cat# 100216
CD3 -APC-F750 (clone 17A2)	BioLegend	Cat# 100247
CD3 -CF633 (clone 17A2) [Conjugated in house]	BioLegend	Cat# 100202
CD4 -CF660 (clone RM4-5) [Conjugated in house]	BioLegend	Cat# 100506
CD8 -BV510 (clone 53-6.7)	BioLegend	Cat# 100752
CD11b -BV510 (clone M1/70)	BD	Cat# 562950
CD11c -BV421 (clone N418)	BioLegend	Cat# 117330
CD11c -AF647 (clone N418)	BioLegend	Cat# 117312
CD11c -eFluor615 (clone N418)	eBioscience	Cat# 42-0114-82
CD11c -BV480 (clone HL3)	BD	Cat# 565627
CD31 -BV421 (clone 390)	BioLegend	Cat# 102424
CD31 -BV480 (clone 390)	BD	Cat# 746260
CD31 -AF488 (clone Mec 13.3)	BioLegend	Cat# 102513
CD45.2 -BV510 (clone 104)	BioLegend	Cat# 109838
CD64 -PE (clone X54-5/7.1)	BioLegend	Cat# 139304
CD64 -CF660c (clone X54-5/7.1) [Conjugated in house]	BioLegend	Cat# 139302
CD169 -CF514 (clone 3D6.112) [Conjugated in house]	BioLegend	Cat# 142402
CD207 -AF488 (clone 929F3.01)	Fisher Scientific	Cat# DDX0362A488
CD207 -AF546 (clone 929F3.01)	Fisher Scientific	Cat# DDX0362A546
CD301b -CF660 (clone URA1) [Conjugated in house]	BioLegend	Cat# 146802
Clec9a -αsheep-CF633 (sheep polyclonal)	R&D	Cat# AF6776
CXCR3 -PE (clone CXCR3-173)	Fisher Scientific	Cat# 12-1831-82
Foxp3 -eF570 (clone FJK-16s)	eBioscience	Cat# 41-5773-82
IFNg -PE (clone XMG1.2)	BioLegend	Cat# 505807
iNOS -AF405 (clone C-11)	Santa Cruz	Cat# sc-7271
IRF4 -CF514; -CF594 (clone IRF4.3E4) [Conjugated in house]	BioLegend	Cat# 646402
Jojo-1 iodide	Invitrogen	Discontinued
Ki67-AF700 (clone B56)	BD	Cat# 561277
Lyve1 -biotin (clone ALY7)	eBioscience	Cat# 13-0443-82
Lyve1 -eFluor570 (clone ALY7)	eBioscience	Cat# 41-0443-82
MHC-II -AF700 (clone M5/114.15.2)	BioLegend	Cat# 107622
MHC-II -AF594 (clone M5/114)	Novus Biologicals	Cat# NBP2-21789AF594

MHC-II -Dy395xl; -Dy396xl; (clone M5/114.15.2) [Conjugated in house]	BioLegend	Cat# 107602
Mtb -anti-rabbit-CF750 (rabbit polyclonal)	Abcam	Cat# Ab905
PCREB -AF488 (clone 87G3)	Cell Signaling	Cat# 9187S
PD1 -agoat-CF750 (goat polyclonal)	R&D	Cat# AF1021
PD-L1 -CF594 (clone MIH5)	eBioscience	Cat# 14-5982-82
pS6 -AF488 (clone 2F9)	Cell Signaling	Cat# 4854S
SIRPα -BV421 (clone P84)	BD	Cat# 624124
SIRPα -CF594 (clone P84) [Conjugated in house]	BioLegend	Cat# 144002
TCF1 -PacBlue (clone C63D9)	Cell Signaling	Cat# 9066S
anti-rabbit-AF750	Invitrogen	Cat# A-21039
SA-AF750	Invitrogen	Cat# S21384
SA-ATTO490LS	ATTO-TEC	Cat# AD490LS-61
anti-GFP-AF488	Invitrogen	Cat# A-21311
